# Cuticle development and the underlying transcriptome–metabolome associations during early seedling establishment

**DOI:** 10.1093/jxb/erae311

**Published:** 2024-07-20

**Authors:** Keting Chen, Rupam Kumar Bhunia, Matthew M Wendt, Grace Campidilli, Colton McNinch, Ahmed Hassan, Ling Li, Basil J Nikolau, Marna D Yandeau-Nelson

**Affiliations:** Department of Genetics, Development & Cell Biology, Iowa State University, Ames, IA, USA; Bioinformatics & Computational Biology Graduate Program, Iowa State University, Ames, IA, USA; Roy J. Carver Department of Biochemistry, Biophysics & Molecular Biology, Iowa State University, Ames, IA, USA; Department of Genetics, Development & Cell Biology, Iowa State University, Ames, IA, USA; Interdepartmental Genetics and Genomics Graduate Program, Iowa State University, Ames, IA, USA; Department of Genetics, Development & Cell Biology, Iowa State University, Ames, IA, USA; Undergraduate Genetics Major, Iowa State University, Ames, IA, USA; Molecular, Cellular, and Developmental Biology Graduate Program, Iowa State University, Ames, IA, USA; Department of Genetics, Development & Cell Biology, Iowa State University, Ames, IA, USA; Undergraduate Data Science Major, Iowa State University, Ames, IA, USA; Department of Biological Sciences, Mississippi State University, Mississippi State, MS, USA; Roy J. Carver Department of Biochemistry, Biophysics & Molecular Biology, Iowa State University, Ames, IA, USA; Interdepartmental Genetics and Genomics Graduate Program, Iowa State University, Ames, IA, USA; Molecular, Cellular, and Developmental Biology Graduate Program, Iowa State University, Ames, IA, USA; Center for Metabolic Biology, Iowa State University, Ames, IA, USA; Department of Genetics, Development & Cell Biology, Iowa State University, Ames, IA, USA; Bioinformatics & Computational Biology Graduate Program, Iowa State University, Ames, IA, USA; Interdepartmental Genetics and Genomics Graduate Program, Iowa State University, Ames, IA, USA; Molecular, Cellular, and Developmental Biology Graduate Program, Iowa State University, Ames, IA, USA; Center for Metabolic Biology, Iowa State University, Ames, IA, USA; RIKEN Center for Sustainable Resource Science, Japan

**Keywords:** Cuticle, cuticular waxes, cutin, early seedling establishment, gene networks, joint transcriptome–metabolome analysis, maize

## Abstract

The plant cuticle is a complex extracellular lipid barrier that has multiple protective functions. This study investigated cuticle deposition by integrating metabolomics and transcriptomics data gathered from six different maize seedling organs of four genotypes, the inbred lines B73 and Mo17, and their reciprocal hybrids. These datasets captured the developmental transition of the seedling from heterotrophic skotomorphogenic growth to autotrophic photomorphogenic growth, a transition that is highly vulnerable to environmental stresses. Statistical interrogation of these data revealed that the predominant determinant of cuticle composition is seedling organ type, whereas the seedling genotype has a smaller effect on this phenotype. Gene-to-metabolite associations assessed by integrated statistical analyses identified three gene networks associated with the deposition of different elements of the cuticle: cuticular waxes; monomers of lipidized cell wall biopolymers, including cutin and suberin; and both of these elements. These gene networks reveal three metabolic programs that appear to support cuticle deposition, including processes of chloroplast biogenesis, lipid metabolism, and molecular regulation (e.g. transcription factors, post-translational regulators, and phytohormones). This study demonstrates the wider physiological metabolic context that can determine cuticle deposition and lays the groundwork for new targets for modulating the properties of this protective barrier.

## Teaser

Integrated metabolomics–transcriptomics analysis indicates that cuticle deposition is associated with plastid development, lipid metabolism, and molecular regulation.

## Introduction

One of the key evolutionary developments that enabled plants to colonize the terrestrial environment appears to have been the ability to synthesize an external hydrophobic barrier, the cuticle (Seale, 2020). Although the primary function of the cuticle is as a water barrier, additional attributes assigned to the cuticle include protection from many biotic (e.g. bacterial, fungal, and insect pests) and abiotic (e.g. drought and UV radiation) environmental stressors (Shepherd and Wynne Griffiths, 2006; Yeats and Rose, 2013).

The cuticle is composed of solvent extractable lipids (i.e. cuticular waxes) infused within and laid on top of a cutin polyester matrix (Kolattukudy and Walton, 1973; Kolattukudy, 1980). The cutin polymer on the aerial organs of terrestrial plants is very similar to the suberin polymer in the endodermis of roots, in that they are both comprised of aliphatic and aromatic monomers including fatty acids, oxygenated fatty acids (e.g. hydroxy-fatty acids, epoxy-fatty acids, and α,ω-dicarboxylic acids), fatty alcohols, glycerols, and phenolic acids (Fernández *et al.*, 2016; Renault *et al.*, 2017; Philippe *et al.*, 2020b). Cutin mainly consists of fatty acids and oxygenated fatty acids, whereas suberin has a higher proportion of fatty alcohols, phenolic acids, and glycerols (Philippe *et al.*, 2020b). The physiological function of roots is to collect water and water-soluble nutrients from the soil matrix, thus the structure and role of suberin in the endodermis of the root is different from that of the cuticle on the aerial organs (Baxter *et al.*, 2009). Specifically, a cuticle is associated only with the initial cap of the developing roots and is not present elsewhere on the roots (Berhin *et al.*, 2019). In contrast, the suberin polymer is associated with lignified Casparian strips and suberin lamellae within the root endodermis, which appear to function as a water barrier that facilitates water movement (Calvo‐Polanco *et al.*, 2021).

Classically, the cuticle was thought to be an independent structure that laid on top of the cell wall. Because accumulating evidence demonstrates that polysaccharides and phenolics are intercalated within the cutin polyester matrix (Pollard *et al.*, 2008; Guzmán *et al.*, 2014a, b, c; Fich *et al.*, 2016; Philippe *et al.*, 2020a; Reynoud *et al.*, 2021, 2022), the cuticle has more recently been considered as a lipidized continuum of the epidermal cell wall (Fernández *et al.*, 2016; Ziv *et al.*, 2018; Philippe *et al.*, 2020b; Reynoud *et al.*, 2021, 2022). In this study, we consider the ester-linked monomers that form the cutin or suberin matrix as monomers that contribute to the lipidized cell wall biopolymers, that is, lipidized cell wall monomers.

The lipidized cell wall contains both aliphatic and phenolic monomers. The aliphatic monomers (e.g. components of cutin and suberin) are generated via the hydroxylation of 16- or 18-carbon chain-length fatty acyl-CoAs, which can occur at multiple positions on the alkyl chain (i.e. at the 2-position, midchain, or at the ω-position), and some of these hydroxy-fatty acids can be converted to epoxy-fatty acids (Fich *et al.*, 2016) and α,ω-dicarboxylic acids (Pollard *et al.*, 2008; Philippe *et al.*, 2020b). The aromatic monomers (e.g. phenolic acids) are produced by phenylpropanoid metabolism (Nawrath, 2002; Pollard *et al.*, 2008; Chen *et al.*, 2022; Woolfson *et al.*, 2022). The cutin and suberin polyesters are then formed by esterification between the hydroxylated-fatty acids and glycerol-3-phosphate (Li *et al.*, 2007) or phenolic acids (Rautengarten *et al.*, 2012), and are transported extracellularly for the final polymerization reaction, which is presumably catalyzed by members of the GDSL-lipase/esterase superfamily, for example, cutin synthase for cutin polymerization (Yeats and Rose, 2013; Fich *et al.*, 2016; Philippe *et al.*, 2020b).

In contrast to cutin and suberin, the cuticular waxes consist of different mixtures of lipid classes mostly derived from very-long-chain fatty acyl-CoAs (VLCFA-CoAs), including very-long-chain fatty acids (VLCFAs), fatty aldehydes, primary and secondary fatty alcohols, wax esters, hydrocarbons, ketones, and terpenes (Fernández *et al.*, 2016). The VLCFA-CoA precursors of the cuticular wax components are products of the endoplasmic reticulum-bound fatty acid elongase system that iteratively adds two-carbon units per elongation cycle to preexisting 16- or 18-carbon fatty acyl-CoAs (Yeats and Rose, 2013; Campbell *et al.*, 2019). Downstream of fatty acid elongase, two parallel pathways generate primary fatty alcohols, fatty aldehydes, and wax esters (the reductive pathway), or aldehydes, hydrocarbons, secondary fatty alcohols, and ketones (the decarbonylative pathway). Notably, these two pathways can utilize VLCFA-CoAs of a variety of chain lengths (20–34 carbons and possibly longer), generating a homologous series of VLCFA derivatives as a mixture of cuticular wax metabolites (Kolattukudy and Walton, 1973; Yeats and Rose, 2013).

Cuticle biosynthesis is developmentally regulated, as manifested by different cuticle compositions among the different organs of a plant or even different tissues within the same organ. For example, in maize, fatty alcohols constitute >60% of the cuticular waxes on leaves of juvenile identity, but they account for only ~15% on adult-identity leaves. These compositional differences are juxtaposed with an increased abundance of wax esters (from ~15% to 40%) and hydrocarbons (from 1% to ~20%) (Avato *et al.*, 1987). More recent studies have revealed that on developing adult-identity maize leaves, the abundance of cutin monomers increases along the length of the leaf. Similarly, the predominant wax components differ according to the position on the leaf, with hydrocarbons being predominant at the leaf base and alkyl esters at the leaf tip (Bourgault *et al.*, 2020). In contrast, the stigmatic silks of maize express a very different composition of cuticular waxes, being rich in both saturated and unsaturated hydrocarbons (often exceeding 90% of the cuticular waxes), with minor amounts of VLCFAs, fatty aldehydes, and fatty alcohols (Perera *et al.*, 2010; Loneman *et al.*, 2017; Dennison *et al.*, 2019; Chen *et al.*, 2024).

In this study, transcriptome profiles coupled with profiles of lipidized cell wall monomers and cuticular waxes were gathered from six seedling organs of four maize genotypes: two agronomically important inbred lines, B73 and Mo17, for which high-quality genome sequences are available (Jiao *et al.*, 2017; S. Sun *et al.*, 2018; Hufford *et al.*, 2021; Chen *et al.*, 2023), and the reciprocal hybrids, B73×Mo17 and Mo17×B73. The six seedling organs represent a spatial gradient that captures the transition of plant growth mode from heterotrophic skotomorphogenesis (i.e. seedling development in darkness) to autotrophic photomorphogenesis (i.e. seedling development in light). The gene networks underlying the changes in cuticle composition during this transition were queried by a multi-omics integration approach designed to assess the joint statistical associations between transcriptomes and metabolomes. This approach identified genetic networks comprising ~1900 genes associated with the deposition of the two different components of the cuticle. These gene networks were composed not only of genes already known to be components of cuticle biosynthetic pathways, but also of many genes, and thereby biological processes, not previously recognized as being associated with cuticle deposition, including post-translational regulation, lipid degradation pathways, and signaling mechanisms.

## Materials and methods

### Plant materials

Seeds of the maize inbred lines B73 and Mo17, and the reciprocal hybrids B73×Mo17 and Mo17×B73, were sown in flats of 16 pots with Redi-Earth soil (Hummert, Earth City, MO, USA). Three biological replicates were grown per genotype, with each replicate grown in its own flat that included eight individual pots (one seed per pot) for each of the four genotypes. Plants were watered every 48 h with a solution supplemented with calcium nitrate at a concentration of 720 g l^–1^ (Fisher, Waltham, MA, USA) and magnesium sulfate at a concentration of 370 g l^–1^ (Fisher). In addition, the soil was treated with Marathon pesticide (Hummert) to control pests. Plants were grown in a climate-controlled greenhouse, maintained at 30% humidity, under a diurnal cycle of 16 h of illumination (at light intensity 230 µmol m^–2^ s^–1^) and 8 h of darkness, at 28 °C and 21 °C, respectively. Plants were harvested for organ dissection based on their height (between 12 cm and 15 cm). Generally, organs from five or six plants per replicate per genotype were sampled and pooled for RNA and cuticle extraction. Coleoptiles were harvested at 6 d after sowing from B73 and Mo17, and at 5 d after sowing from the reciprocal hybrids. The other seedling organs (roots, sheath of first leaf, first leaf blade, encased leaves, and second leaf blade) were harvested at 9 d after sowing from B73 and Mo17, and at 8 d after sowing from the reciprocal hybrids. Pools of each of these tissues were flash frozen in liquid nitrogen and stored at –80 °C.

### Cuticular wax extraction and quantification

Pooled tissue samples were separately dipped for 30 s at room temperature into 10 ml of chloroform containing 5 μg of heptadecanoic acid (Sigma, St. Louis, MO, USA) as the internal quantification standard. The extracts were dried under a stream of nitrogen gas and derivatized by incubation at 70 °C for 30 min with 100 μl of *N*,*O*-bis (trimethylsilyl) trifluoroacetamide (BSTFA) and 100 μl anhydrous pyridine (Sigma). After the derivatization reaction, excess BSTFA and pyridine were evaporated under a stream of nitrogen gas, and the residue was dissolved in 500 μl of heptane:toluene (1:1, v/v) and subjected to analysis by gas chromatography-mass spectrometry (GC-MS). Cuticular wax metabolites were identified using AMDIS software (Stein, 1999) with the NIST14 mass spectral library (http://nistmassspeclibrary.com) and metabolite concentrations were calculated relative to the internal standard and presented as µmol^.^g^–1^ dry weight.

### Extraction and quantification of lipidized cell wall polymeric material

Using a previously developed method for isolating cutin (Bhunia *et al.*, 2018), the lipidized cell wall biopolymers were extracted from aerial and root organ samples. The recovered polymeric material was highly enriched in cutin (isolated from the aerial organs) or suberin (isolated from roots), and following depolymerization by transmethylation, the recovered lipidized cell wall monomers were analyzed by GC-MS.

These extracts of the lipidized cell wall biopolymers were prepared in parallel from aliquots of the same pooled tissue samples that were used for cuticular wax analysis. Dried tissue (10–20 mg per sample) was exhaustively delipidated by overnight immersion at room temperature in isopropanol containing 0.01% (w/v) butylated hydroxytoluene (BHT) in PTFE screw-capped glass tubes on a rotatory shaker. The solvent was discarded the next day, and the tissue was extracted with two aliquots of chloroform:methanol (2:1) containing 0.01% (w/v) BHT, and subsequently with an aliquot of methanol containing 0.01% (w/v) BHT. Each of these extractions was for a period of 2 h at room temperature. Finally, the methanol was discarded and the delipidated residue preparation was dried by lyophilization to a constant dry weight.

Acid-catalyzed transmethylation was conducted by adding to the dry residue 2 ml of freshly prepared methanolic sulfuric acid (4% v/v) and 0.2 ml of toluene, along with 10 μg of heptadecanoic acid as an internal quantification standard. The mixture was heated at 80 °C for 2 h and, after cooling, the mixture was extracted with 4 ml dichloromethane and 1 ml of 0.9% (w/v) NaCl in 100 mM Tris–HCl buffer, pH 8.0. The non-polar phase was separated from the polar phase by centrifugation at 400 *g* for 2 min, and the two phases were recovered separately and dried. Each sample was silylated at 70 °C for 30 min with 100 μl anhydrous pyridine and 100 μl BSTFA. The mixture was subsequently dried under a stream of nitrogen gas, and the residue was dissolved in 500 μl of heptane:toluene (1:1, v/v) and subjected to GC-MS analysis.

Methylated and silylated cuticular wax constituents and lipidized cell wall monomers were analyzed using an Agilent Technologies Model 7890A gas chromatograph coupled to a Model 5975C mass spectrometer (Agilent Technologies, Santa Clara, CA, USA). The GC conditions were as follows: the inlet temperature was held constant at 280 °C, the helium carrier gas was at a constant flow rate of 1 ml min^–1^ through an Agilent 122-0112 DB-1ms column (15 m × 250 µm × 0.25 µm). The oven temperature was initially set at 70 °C and then increased by 10 °C min^–1^ to 340 °C and held at that temperature for 6 min; the transfer line was set to 280 °C.

The detection and quantification of individual monomers was accomplished using an Agilent Model 5975C mass spectrometer under standard conditions with 280 °C ion source. Absolute quantification was determined by comparing the ion signal of each peak to that of the heptadecanoic acid internal standard.

### Liquid chromatography–mass spectrometry analyses

The efficacy of the delipidation processes that removed intracellular lipids from the extracellular polymeric lipid preparations was assessed by liquid chromatography with tandem mass spectrometry (LC-MS/MS) analyses. Delipidated cuticular polymeric preparations, collected from the first and second leaves of three seedlings, were subjected to LC-MS/MS analyses, which identified such intracellular lipids as phosphatidylethanolamine, phosphatidic acid, diacylglycerol, and triacylglycerol (identified in positive mode), and free fatty acids (identified in negative mode). These analyses indicated that delipidation resulted in the removal of 93–98% of the intracellular lipids (Supplementary Table S1;  Supplementary Fig. S1).

Specifically, lipids extracted from the delipidated samples were subjected to LC-MS/MS analysis using instruments maintained by the W.M. Keck Metabolomics Research Laboratory (https://www.biotech.iastate.edu/metabolomics/) in both positive and negative ionization modes using previously established protocols (Kimbara *et al.*, 2013; Okazaki *et al.*, 2015). LC separation was performed with a 1290 Infinity Binary Pump UHPLC instrument (Agilent Technologies), equipped with a Waters Acquity UPLC HSS T3 (1.8 μm × 1.0 mm × 50 mm) analytical column (Waters Corporation, Milford, MA, USA), coupled to an Agilent Technologies 6540 UHD Accurate-Mass Q-TOF mass spectrometer (Agilent Technologies). A 1.4 µl aliquot of each sample was injected into the LC system. Chromatography was carried out at 55 °C at a flow rate of 0.15 ml min^–1^. The binary solvent system comprised a step gradient of Solvent A and B. Solvent A was composed of acetonitrile:water:1 M ammonium acetate:formic acid (158 g:800 g:10 ml:1 ml) and Solvent B was composed of acetonitrile:2-propanol:1 M ammonium acetate:formic acid (79 g:711 g:10 ml:1 ml). The solvent gradient conditions were as follows: 35% Solvent B at 0 min, 70% Solvent B at 3 min, 85% Solvent B at 7 min, 90% Solvent B at 10 min, 90% Solvent B at 12 min, and 35% Solvent B at 12.5 min. A 6 min post-run of 100% Solvent A was conducted after each LC-MS/MS data acquisition cycle. All solvents were LC-MS grade (Fisher Scientific, Waltham, MA, USA).

Metabolites were detected using electrospray ionization in both negative and positive ionization modes. Nitrogen was used as the service gas for the ion source, with a drying gas flow rate of 11 l min^–1^ at a temperature of 350 °C, a nebulizing pressure of 158.6 kPa, and a sheath gas flow of 11 l min^–1^ at 400 °C. The capillary was maintained at 4000 V. The mass spectrometer was operated in high-resolution mode (4 Gz) with a scan range from *m/z* 100 to *m/z* 1700. An acquisition rate of 2.0 spectra per second was used. Reference masses were monitored for continuous mass calibration during LC-MS data acquisition. In positive mode these were *m/z* 121.050873 and *m/z* 922.009698, and in negative mode they were *m/z* 112.985587 and *m/z* 1033.988109. MS/MS spectra were captured using a collision energy of 30 V. Data evaluation and peak detection were performed using Agilent MassHunter Qualitative Analysis (version 10.0) and Mass Profiler programs (version 8.0) (Agilent Technologies). Metabolite peaks were identified using accurate mass spectral analysis by comparison to the METLIN database library (Smith *et al.*, 2005), with additional identification performed using the NIST20 spectral library (Agilent Technologies).

### RNA extraction and sequencing

RNA was extracted from pooled tissues by the TRIzol method (Invitrogen, Carlsbad, CA, USA) and subsequently purified using a Qiagen RNeasy MinElute Cleanup Kit. RNA sequencing (RNA-seq) libraries for each biosample were prepared using a KAPA Stranded RNA-Seq Library Preparation Kit (Illumina, San Diego, CA, USA) and subjected to RNA-seq at the Beijing Genomics Institute. The 100-cycle paired-end sequencing data were acquired from three lanes of Illumina HiSeq 4000, with each lane containing 24 biosamples comprising six seedling organs from four genotypes.

Preprocessing of resultant RNA-seq reads for removal of adapter sequences and subsequent data quality control was performed by FastQC as described in McNinch *et al.* (2020), revealing an average read length of 100 bp and a median read depth of 39.1 million reads per biological replicate. Reads from all samples were aligned to the B73 genome (Jiao *et al.*, 2017) that already incorporated Mo17 variants (S. Sun *et al.*, 2018) into the genome build, prior to aligning by the HISAT2 alignment program (D. Kim *et al.*, 2015). Next, transcripts were assembled and quantified on a fragments per kilobase of transcript per million mapped reads (FPKM) basis as described in McNinch *et al.* (2020).

### Statistical analysis of cuticle metabolomes

The effects of seedling genotype, organ, and the genotype × organ interaction on the abundance of each class of lipidized cell wall monomers or cuticular waxes was evaluated by type I analysis of variance (ANOVA). Post-hoc Tukey’s honest significant difference (HSD) tests were applied to further examine the genotype differences within each organ. The ANOVA and HSD tests were conducted using JMP® (Version 15.0, SAS Institute Inc., Cary, NC, USA).

### Visualization of metabolomics and transcriptomics data

Prior to principal component analysis (PCA) and *t*-distributed stochastic neighbor embedding (tSNE) visualization, metabolomics and transcriptomics datasets were transformed such that the average expression of individual genes or metabolites was 0, with the corresponding variance set to a value of 1. For each dataset, PCA was performed using the princomp() function in the R/stats package (R Core Team, 2019), and tSNE visualization, based on all principal components (PCs), was performed using the Rtsne() function in the R/Rtsne package (Krijthe, 2015).

### Gene co-expression network analysis

The co-expressed gene clusters were determined by weight gene co-expression network analysis (WGCNA) using the R/WGCNA package (Langfelder and Horvath, 2008) as described in McNinch *et al.* (2020). Transcriptome datasets with the expression profiles of 30 931 genes were first subjected to data filtering by WGCNA/goodSampleGenes(), which left 22 841 genes expressed in >50% of the total samples and ready for analysis by WGCNA. Briefly, first, an adjacency matrix among the expressed genes was derived from biweight midcorrelations with a soft-threshold power of 16. Next, a topographical overlap matrix was constructed based on the adjacency matrix and used for the hierarchical clustering conducted using hclust(). Co-expressed gene clusters were refined from the resultant dendrogram using the function cutreeDynamic() followed by mergeCloseModules(). An eigengene value was computed for each cluster to represent the overall expression pattern of all genes in the cluster. A second hierarchical clustering and Pearson correlation analysis was performed among the cluster eigengenes to classify clusters with similar expression patterns into separate classes. Each class comprised clusters with correlation coefficients greater than 0.5.

Finally, hub genes were identified for each co-expression cluster. A gene was considered as a cluster hub gene if it satisfied the following three requirements: (i) an above-average significant connection degree (Das *et al.*, 2017), that is, it is connected in its expression pattern with a large number of other genes in the cluster; (ii) the correlation value between the candidate hub gene and the corresponding eigengene is >0.8; and (iii) the correlation between the candidate hub gene and traits of interest (i.e. tSNE components for metabolomics data) is greater than an assigned cutoff value (Liu *et al.*, 2019). Different correlation cutoffs were selected for each cluster such that ~5% of the genes in a cluster were identified as hub genes.

### Multi-omics integration pipeline: gene-to-metabolite associations

Partial least squares regression (PLS), sparse partial least squares regression (sPLS), and random generalized linear model (rGLM) were used to identify candidate genes with different levels of expression in the different seedling organ samples. Data centering was applied in PLS to set the average of individual metabolite concentration or gene expression to a value of 0. Data scaling for sPLS and rGLM was performed so that the variance of metabolite concentration or gene expression was 1. PLS and sPLS were conducted using scripts that were implemented in R, and rGLM was conducted using the R/randomGLM package (Song *et al.*, 2013), with modifications to the original scripts (https://github.com/ketingchen/MultiOmicsIntegration.git).

Two types of response variables were independently used to represent the compositional changes in the cuticle metabolome: (i) the concentration profiles of all metabolites (PLS and sPLS) and (ii) single-value tSNE components that captured the major variations across metabolomes. The PLS and sPLS statistical methods utilized the former type of responses, whereas rGLM used the latter, because this method can incorporate only a single-value response variable.

In each of these models, an importance score, S,  was calculated to represent the contribution of individual genes in predicting metabolome composition. The probability of obtaining a background importance score, $S_{B}$, exceeding or equaling S ($P\left(S_{B}\geq S\right))\;$ in datasets devoid of transcriptome–metabolome correlations was evaluated by a permutation test that is described in Supplementary Protocol S1. A gene was selected as a putative cuticle-related gene by a model when S>1 and $P\left(S_{B}\geq S\right)$<0.01.

This suite of multivariate statistical methods was applied separately to interrogate the gene-to-lipidized cell wall monomer and gene-to-cuticular wax constituent associations. An individual expressed gene was considered as a putative cuticle-related gene impacting the composition of the lipidized cell wall monomer and/or cuticular waxes if it was selected by at least one of the three statistical approaches.

### Multi-omics integration pipeline: gene cluster-to-metabolite associations

The association between a co-expressed gene cluster and cuticle metabolomes was interrogated by a random forest (RF) regression model that incorporated the eigengene expression for each cluster and the expression data of non-clustered individual genes as the predictors, and the tSNE components for lipidized cell wall monomers or cuticular waxes as the response variables. Three metrics were used to evaluate the importance of a gene cluster (or an individual gene) on the metabolome compositions: (i) predictive *R*^2^ (i.e. 1–sum of squares error/total sum of squares), (ii) root mean square error, and (iii) mean absolute percentage error (100×|predicted value–actual value|/actual value). In detail, for each predictor, two RF models were constructed, one including the full set of predictors and the other having one specific predictor removed. The difference in each evaluating metric between the two models was subsequently calculated. A bootstrapping strategy was used to calculate the *P*-values associated with the difference, which were later corrected among all predictors to control the false discovery rate <5%. A predictor was significantly associated with the compositional changes of lipidized cell wall monomers and/or cuticular waxes if removal of this predictor from the RF model reduced the model performance in at least two metrics, with *P*-values <0.01. The RF model was performed by using the R/randomForest package (Liaw and Wiener, 2002) and the bootstrapping strategy that evaluates the predictor importance and computes the *P*-values was performed using scripts that were implemented in R (https://github.com/ketingchen/MultiOmicsIntegration.git).

### Gene functional enrichment analysis

Gene Ontology (GO) enrichment tests for the biological process domain were performed using the R/topGO package (Alexa and Rahnenfuhrer, 2021) and maize-GAMER GO annotations (Wimalanathan *et al.*, 2018), as described in McNinch *et al.* (2020). The resultant *P*-values were corrected to control the false discovery rate <5%. GO terms with corrected *P*-values <0.0001 were considered as significantly enriched. The significant GO terms were then trimmed by REVIGO, a web server tool that removes functional redundancy in each list of GO terms via the identification of the most informative representative terms according to ‘parent–child’ hierarchy and semantic similarity among GO terms (Supek *et al.*, 2011).

Annotations of MapMan pathway categories (i.e. function bins) for all protein-coding maize genes were derived from the maize B73 draft genome version 4, as retrieved from the MapMan website (https://mapman.gabipd.org/mapman). MapMan-based function enrichment tests were performed by Fisher’s exact test. The resultant *P*-values were corrected to control the false discovery rate <5%. MapMan bins with corrected *P*-values <0.05 were considered as significantly enriched.

The putative cuticle-related genes identified by multi-omics integration analysis and annotated with significantly enriched GO terms and MapMan bins were visualized in Cytoscape (version 3.8.2) (Shannon *et al.*, 2003) to present their co-expression clustering membership that was determined by WGCNA.

## Results

### Cuticular wax composition of seedling organs from different genetic backgrounds

**Fig. 1.**
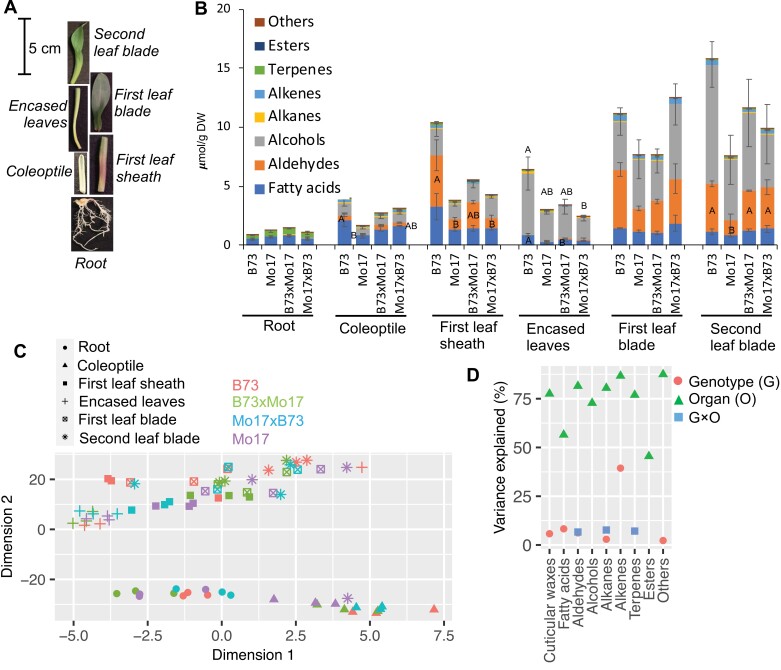
Comparison of cuticular waxes among seedling organs from two maize inbred lines, B73 and Mo17, and their reciprocal hybrids. (A) Seedling organs collected for metabolomics and transcriptomics analyses. (B) Accumulation of cuticular wax lipid classes among the organs of different genotypes. Different letters indicate statistically significant differences in the accumulation of different cuticular wax lipid classes among different genotypes for a given organ (*P*<0.05; Tukey’s HSD test). (C) *t*-Distributed stochastic neighbor embedding visualization of different cuticular wax metabolomes expressed by triplicate analysis of different organs from different genotypes (D) Impact of genotype, organ, and genotype × organ interactions on different cuticular wax lipid classes, calculated as partial *R*^2^ values by type I ANOVA. Factors that did not significantly impact variation in the cuticular wax lipid classes (*P*<0.05) are not shown.

Cuticular waxes were profiled from six seedling organs [roots, coleoptiles, first leaf sheaths, leaves encased by the first leaf sheath (encased leaves), and the first and second leaf blades] (Fig. 1A) of four different maize genotypes (the two inbred lines, B73 and Mo17, and the reciprocal hybrids, B73×Mo17 and Mo17×B73). These analyses identified six classes of cuticular wax components: VLCFAs, fatty alcohols, fatty aldehydes, wax esters, hydrocarbons, and a small quantity of terpenes, the latter being particularly abundant in roots (Fig. 1B). The alkyl cuticular wax components ranged between 12 and 34 carbon atoms in chain length, with the predominant chain length being 32 carbons for fatty acids, fatty alcohols, and fatty aldehydes, and 31 carbons for alkanes and alkenes (Supplementary Fig. S2A; Supplementary Table S2A). The ranges of chain lengths, however, were slightly different among the different classes of cuticular wax components (e.g. fatty alcohols ranged between 18 and 34 carbon atoms, whereas the fatty aldehydes ranged between 26 and 34 carbon atoms) (Supplementary Fig. S2A; Supplementary Table S2A).

Regardless of the genotype that was evaluated, the first and second leaf blades accumulated the largest amount of cuticular waxes, whereas the first leaf sheath and the encased leaves accumulated approximately half that amount; minimal accumulation of cuticular wax-like metabolites was observed for the roots. Furthermore, the majority of the cuticular waxes that were recovered from these aerial organs were fatty alcohols and fatty aldehydes, which together accounted for ~80% of the recovered cuticular wax components (Fig. 1B). The 32-carbon fatty aldehyde was the predominant cuticular wax constituent identified in first and second leaf blades, whereas it was only a minor constituent in coleoptiles, first leaf sheaths, encased leaves, and roots (Supplementary Table S2A). In addition, the cuticular wax compositions of roots and coleoptiles differed substantially from those of the other organs, comprising cuticular wax metabolites of shorter chain lengths than for the other organs (i.e. chain lengths of 30 carbons or shorter; Supplementary Fig. S2A). Cuticular wax-like metabolites accumulated to much lower concentrations on the roots, and comprised approximately equal amounts of terpenes and VLCFAs. In contrast, cuticular wax metabolites accumulated to higher concentrations on coleoptiles and consisted primarily of VLCFAs and fatty alcohols (Fig. 1B).

The relative contributions of different genotypes and organ types to the variation in cuticular wax composition was visualized via two unsupervised statistical methods, PCA and tSNE (Fig. 1C; Supplementary Fig. S3A). Both visualization methods indicated that the differences in cuticular wax metabolomes are primarily determined by organ type, with minor contributions determined by genotype (Fig. 1C). Specifically, PC1 and PC2, which together explained 55% of the observed variance in cuticular wax composition, separated the root and coleoptile samples from the other seedling organs. Examination of each individual class of metabolites by ANOVA revealed that the organ type explained a large proportion of the observed variance for most cuticular wax classes (ranging between 45% and 88%), and genotype and genotype × organ interactions contributed to a far lesser extent (<10%) (Fig 1D). Collectively, these analyses demonstrate that the major influence on the cuticular wax metabolome is the seedling organ type rather than the genetic background of the seedling.

### Lipidized cell wall monomer composition of seedling organs from different genetic backgrounds

In parallel to the analyses of the cuticular waxes, the six dissected seedling organs were also analyzed to determine the monomer compositions of lipidized cell wall biopolymers among the inbred lines and their hybrids. Thorough delipidation of the tissues was required prior to monomerization of the lipidized cell wall biopolymers and, as detailed in the Materials and methods, the delipidation process removed 93–98% of the intracellular lipids (Supplementary Fig. S1; Supplementary Table S1).

**Fig. 2.**
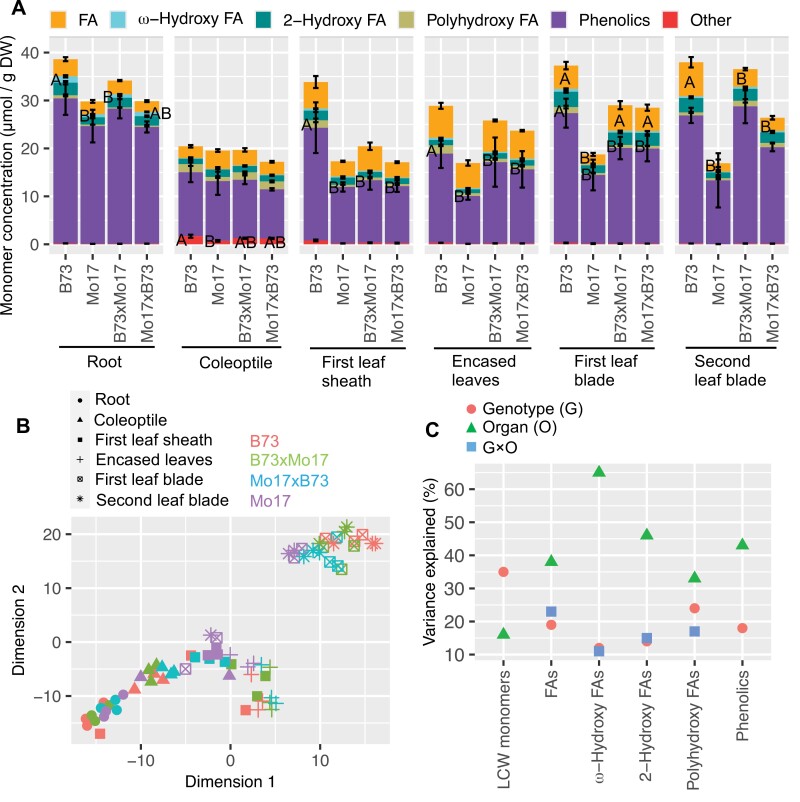
Comparison of lipidized cell wall (LCW) monomers among seedling organs from maize inbred lines and reciprocal hybrids. (A) Accumulation of LCW monomers among the organs of different genotypes. Different letters indicate statistically significant differences in the accumulation of different lipidized cell wall monomer classes among different genotypes for a given organ (*P*<0.05; Tukey’s HSD test). (B) t-Distributed stochastic neighbor embedding visualization of LCW monomer metabolomes expressed by triplicate analysis of different organs from different genotypes. (C) Partial *R*^2^ values representing the impact of genotype, organ, and genotype × organ interaction on different monomer classes as evaluated by type I ANOVA. Factors without significant (*P*<0.05) impact are not shown.

Chemical depolymerization of these biopolymeric materials identified five major monomer subclasses: phenolics, fatty acids, and three types of hydroxy-fatty acids, with the hydroxyl group situated at either the 2-position or the ω -position, as well as di- and tri-hydroxy-fatty acids (Fig. 2A; Supplementary Table S2B). The phenolic compounds that were recovered included caffeic acid, coumaric acid, and ferulic acid, which could be derived from the cutin or suberin polyesters or from other ester-linked polymeric components of the cell wall. Over 50% of the recovered fatty acids consisted of the unsaturated fatty acid 18:2, and the rest were saturated fatty acids with acyl chain lengths ranging between 16 and 30 carbons. Although the 18:2 fatty acid monomer could be derived from membrane lipids, a comparison of its abundance in the delipidized and non-delipidized samples suggested that this fatty acid is not membrane lipid-associated (Supplementary Tables S1A, S2B). The ω-hydroxy fatty acids included saturated and unsaturated fatty acids ranging between 16 and 22 carbons, 2-hydroxy fatty acids included 16:1 fatty acid and the saturated fatty acids ranging between 22 and 26 carbons, and polyhydroxy fatty acids were derived from 18:0 fatty acid (Supplementary Fig. S2B; Supplementary Table S2B).

PCA and tSNE analyses were used to visualize the effects of different genotypes and organ types on the variance of the lipidized cell wall monomer compositions (Fig. 2B; Supplementary Fig. S3B). In combination, PC1 and PC2 explained 50% of the observed variance in monomer composition and separated the root and coleoptile samples from the other seedling organs (Supplementary Fig. S3B). The higher-resolution tSNE visualization generated a more distinct separation of the two leaf blade samples relative to the other seedling organs (Fig. 2B). ANOVA supported the conclusions reached by the PCA and tSNE data visualizations, namely, that different seedling organs explained 16–65% of the observed variance among the lipidized cell wall monomer classes, while the different genotypes among the samples had a smaller, but still substantial impact, explaining 12–35% of the observed variance in each of the metabolite classes. Finally, the interaction between genotype and organ type explained 11–23% of the observed variance (Fig. 2C). Collectively, these analyses demonstrate that, analogous to the cuticular wax metabolite profiles, organ type predominantly influences lipidized cell wall monomer composition, while genotype has a smaller effect.

### Variation in global gene expression among seedling organs of different genotypes

The transcriptome of each seedling organ was sequenced, and the resulting transcripts were mapped to the B73 reference genome (Jiao *et al.*, 2017) incorporating the corresponding gene sequence variants from Mo17 (S. Sun *et al.*, 2018). These assemblies were used to quantify the expression of 30 931 genes that were detected in the six seedling organs from the four genotypes that were evaluated. Visualization of the datasets by tSNE and PCA demonstrated that the root transcriptomes segregated into a discrete cluster and the transcriptomes of the other organs were grouped into three distinct clusters, one containing the coleoptile and first leaf sheath, another containing the first and second leaf blades, and the third cluster encompassing the encased leaves (Supplementary Fig. S4). However, the transcriptomes within these clusters exhibited no consistent separation based on genotype.

Differentially expressed gene (DEG) analysis was performed to compare the transcriptomes between every pair of genotypes (i.e. six comparisons) for each organ. The total non-redundant DEGs between every pair of genotypes (Supplementary Fig. S5) comprised <8% of the entire transcriptome. The majority of the DEGs identified in each organ occurred between the inbred lines B73 and Mo17, and between the combined hybrids and one of the parental inbred lines, whereas no DEGs were detectable between the hybrids B73×Mo17 and Mo17×B73 (Supplementary Fig. S5). In contrast, the total non-redundant DEGs between every pair of organs comprised 34–46% of the entire transcriptome in each genotype (Supplementary Fig. S6). Collectively, these data demonstrate that the gene expression program is primarily driven by the developmental program that generates the six different organs, and that these programs are similar among the genotypes that were assessed.

### Integrated analysis of organ transcriptomes and cuticle metabolomes

**Fig. 3.**
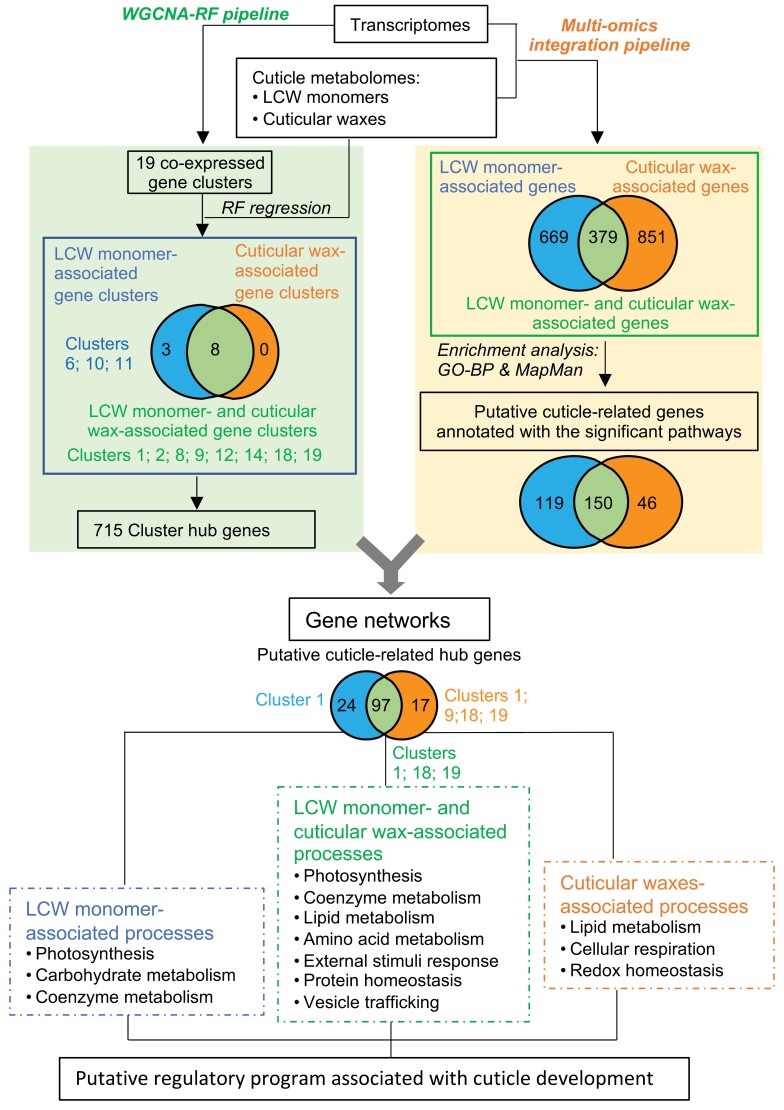
Schematic summary of the outcomes from the parallel analysis of the combined transcriptome–metabolome dataset by two independent pipelines. The weighted gene co-expression network analysis–random forest (WGCNA-RF) pipeline identified 19 co-expression gene clusters, of which three were associated with the accumulation of lipidized cell wall (LCW) monomers and eight were associated with the accumulation of both LCW monomers and cuticular waxes. The multi-omics integration pipeline identified 119, 46, and 150 genes associated with LCW monomers, cuticular waxes, and the combination of LCW monomers and cuticular waxes, respectively. Integrating these two outcomes generated gene networks that identified different metabolic pathways associated with different processes that assemble the integrated cuticle.

In the context of different genotypes and different seedling organs, two independent approaches were used to interrogate the associations between the transcriptome and cuticle composition. These are schematically illustrated in Fig. 3, and consisted of: (i) a WGCNA-RF pipeline that assessed the relationship between co-expressed gene clusters and the changes in cuticle composition, and (ii) a multi-omics integration pipeline that conducted a joint statistical association analysis between individual transcripts and individual cuticle metabolites. The first of these pipelines identified gene clusters with significant collective association with cuticle composition, while the second pipeline identified individual genes associated with differences in cuticle composition. Because each pipeline identified a large number of genes potentially related to cuticle deposition, the results from the two pipelines were integrated to refine the gene networks that likely underlie the observed changes in cuticle composition among seedling organs and between genotypes (Fig. 3).

### WGCNA-RF-based identification of co-expression gene clusters associated with lipidized cell wall monomer and cuticular wax composition

It is often possible to infer that genes within a co-expression cluster that is highly correlated to a trait (in this case a cuticle composition trait) have a significant role in the biological processes that determine that trait (Carlson *et al.*, 2006). Hence, WGCNA was applied to the transcriptome datasets to first reveal clusters of genes that showed co-expression patterns among the six seedling organs from the four genotypes. Subsequently, the application of an RF regression model revealed the association of each co-expressed gene cluster with changes in the composition of the cuticle metabolome. Hub genes within these pre-selected co-expression clusters were specifically identified as the genes showing the strongest connection with cuticle composition (Supplementary Table S3A).

**Fig. 4.**
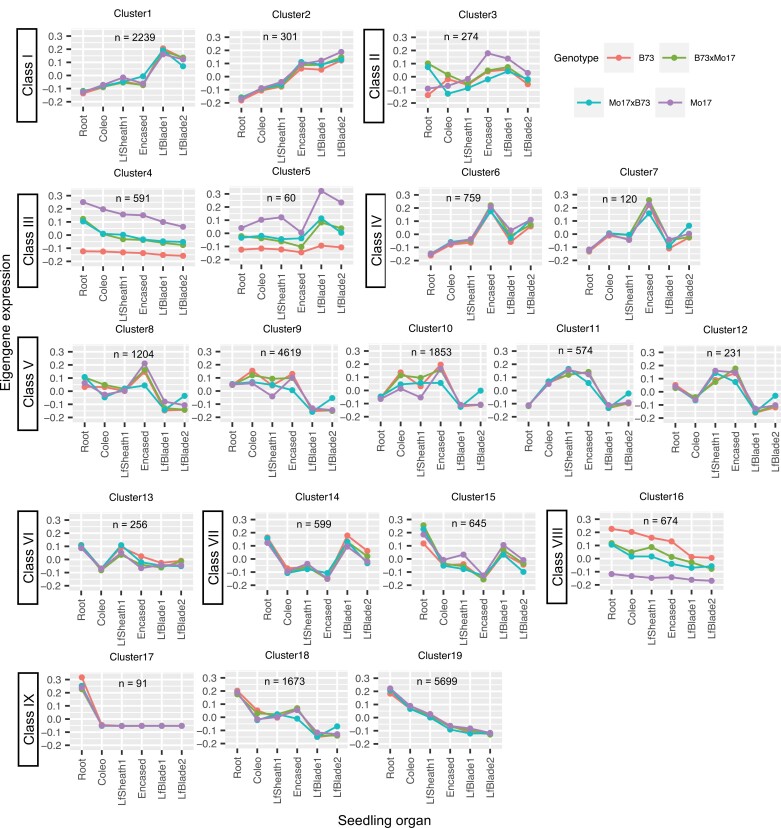
Co-expression gene clusters determined by weighted gene co-expression network analysis. Each color identifies the eigengene expression pattern that represents the co-expressed gene cluster for each maize genotype (i.e. B73, Mo17, B73×Mo17, and Mo17×B73). Hierarchical clustering based on the eigengene expression pattern categorized the 19 co-expression gene clusters into 9 classes (Class I to Class IX). Coleo, coleoptile; Encased, encased leaves; LfBlade1, first leaf blade; LfBlade2, second leaf blade; LfSheath1, first leaf sheath; *n*, number of genes in each co-expression gene cluster.

WGCNA identified 19 co-expression gene clusters encompassing 22 463 genes, leaving only 378 genes that showed expression patterns that were not clusterable (Fig. 4; Supplementary Table S3B). Subsequently, the expression of an eigengene was calculated for each co-expression gene cluster. In each cluster, these eigengenes represented the average gene expression profile per genotype and thereby visualized the cluster average expression pattern among the six organs. Hierarchical clustering further categorized the 19 different co-expression clusters into nine classes (Class I–IX; Supplementary Fig. S7; Fig. 4). Genes in six of these classes (I, IV, V, VI, VII, and IX) exhibited an expression pattern that varied primarily among seedling organs, with little difference among the four genotypes that were evaluated. Genes within Class I clusters showed the highest expression levels in the aerial organs of the seedling, particularly in the leaf blades, whereas Class IX genes showed the opposite pattern, with maximal expression occurring in roots, and minimal expression in the aerial organs. Genes in Classes IV, V, and VI showed maximal gene expression in coleoptiles, first leaf sheaths, or encased leaves, whereas Class VII genes demonstrated minimal expression in these organs.

In contrast, genes in the Class II, III, and VIII clusters exhibited expression patterns that differed among the organs and were also affected by the genotype of these organs. Collectively, these three classes contained only 1599 genes (~7% of the genes analyzed by WGCNA). Opposite effects of genotype were observed between Class III and Class VIII genes, with the expression in B73 being lower than in Mo17 for Class III genes, whereas the expression of Class VIII genes was higher in B73; in both cases, the expression in the reciprocal hybrids was indistinguishable from each other and was at the midpoint between the two parental inbred lines. Finally, the expression of genes in Class II demonstrated a complex interaction between genotype and seedling organs, best illustrated by comparing the expression patterns in roots with the patterns observed in the aerial organs. In roots, higher expression was observed in the two hybrids as compared with the parental lines, but in the aerial organs higher expression was observed in the Mo17 parental line, whereas expression was at a similar, lower level among the other three genotypes (Fig. 4).

The associations between specific gene co-expression clusters and differences in lipidized cell wall monomer and cuticular wax compositions were interrogated by an RF regression model. This model used the calculated expression of the eigengenes that represented each co-expression gene cluster, as well as the experimentally measured expression of the 378 individual genes that were not clusterable, to predict the previously calculated tSNE scores for cuticular waxes (Fig. 1C) and lipidized cell wall monomers (Fig. 2B). Thereby, these analyses identified gene co-expression clusters that are associated with different cuticle compositions. The importance of each co-expression cluster to the model was evaluated by RF model comparisons described in the Materials and methods. These analyses identified 11 co-expression gene clusters as significantly associated with differences in cuticle composition (i.e. Clusters 1, 2, 6, 8, 9, 10, 11, 12, 14, 18 and 19). Eight of these clusters (Clusters 1, 2, 8, 9, 12, 14, 18 and 19) were significantly associated with differences in both cuticular wax and lipidized cell wall monomer composition, and the other three clusters (Clusters 6, 10, and 11) were uniquely associated with differences in lipidized cell wall monomer composition; no clusters were uniquely associated with differences in cuticular wax composition (Fig. 3; Table 1; Supplementary Table S3C, D). Next, we used two criteria to find ‘hub genes’ that were representative of these 11 co-expression gene clusters associated with differences in cuticle composition: (i) high connectivity to the other genes in the cluster (i.e. gene-to-gene associations) and (ii) strong association with the compositional differences in lipidized cell wall monomer and/or cuticular wax traits. A total of 715 hub genes were thus identified that we hypothesize play important roles in determining cuticle composition (Table 1; Supplementary Table S3A). These hub genes include >20 transcription and post-translational regulators, such as bHLH and MADS family transcription factors, and components of the ubiquitin–26S proteasome system (Supplementary Table S3A). Many of these transcriptional regulators have been demonstrated to be key components of gene regulatory networks involved in plant stress responses (X. Sun *et al.*, 2018; Castelán-Muñoz *et al.*, 2019; Doroodian and Hua, 2021).

**Table 1.** Co-expressed gene clusters associated with cuticle composition

Cluster	Number of genes	Associated cuticle fraction^*a*^	Number of putative cuticle-related genes	Gene-to-tSNE correlation threshold^*c*^	Number of hub genes	Number of hub genes that are putative cuticle-related genes
*1* ^*b*^	2239	LCW monomer; Cuticular wax	437^*b*^	0.75	140	131
*2*	301	LCW monomer; Cuticular wax	26	0.7	15	10
*6*	759	LCW monomer	12	0.6	35	1
*8*	1204	LCW monomer; Cuticular wax	6	0.5	58	0
*9*	4619	LCW monomer; Cuticular wax	139	0.65	131	41
*10*	1853	LCW monomer	13	0.55	122	0
*11*	574	LCW monomer	17	0.45	15	1
*12*	231	LCW monomer; Cuticular wax	1	0.3	7	0
*14*	599	LCW monomer; Cuticular wax	3	0.3	5	0
*18*	1673	LCW monomer; Cuticular wax	107	0.65	108	52
*19* ^*b*^	5699	LCW monomer; Cuticular wax	1003^*b*^	0.75	185	179

a Weighted gene co-expression network analysis-based co-expression clusters are listed as being significantly associated with either or both lipidized cell wall (LCW) monomer and cuticular wax compositions. Evaluation of the association between gene clusters and metabolome compositions is detailed in the Materials and methods.

b Clusters that are significantly enriched (corrected *P*-value <0.01 according to Fisher’s exact test) with the putative cuticle-related genes selected by the multi-omics integration analysis.

c Different thresholds for the correlations between individual gene expression and *t*-distributed stochastic neighbor embedding (tSNE) scores of LCW monomers and/or cuticular waxes are selected for each module such that ~5% of genes are identified as hub genes.

### Multi-omics integration-based identification of individual cuticle-related genes associated with lipidized cell wall monomer and cuticular wax composition

Synergistic to the WGCNA-RF approach that identified hub genes, a complementary series of gene–metabolite association approaches were implemented to identify putative cuticle-related genes that may mediate cuticle formation. Specifically, three multivariate statistical models were employed for joint statistical analysis of the metabolome and transcriptome datasets, which aimed to identify individual genes whose expression patterns are statistically associated (corrected *P*-value <0.01) with the accumulation patterns of lipidized cell wall monomers and cuticular wax metabolites. The resultant gene list was abridged by keeping only genes annotated with significantly enriched metabolic pathways (Fig. 3).

The three multivariate statistical models comprised PLS, sPLS, and rGLM. The PLS model identified genes that were highly expressed (i.e. >100 FPKM), whereas the sPLS and rGLM models identified genes with lower expression levels (i.e. >0 and <100 FPKM). In the PLS and sPLS models, the concentrations of all individual lipidized cell wall monomers or cuticular wax metabolites were used as the response variables, whereas in the rGLM, the tSNE components of these metabolites were used as the response variables (Figs 1C, 2B). The resultant list of putative cuticle-related genes (i.e. genes statistically associated with cuticle compositions) comprised ~1900 genes that were identified by any single statistical model (listed in Supplementary Table S4). This gene list contains >100 genes that are transcription factors or potential transcription regulators such as DNA/RNA-binding proteins and components of the ubiquitin–26S proteasome system (Supplementary Table S4). Moreover, this gene list includes 669 genes that are uniquely statistically associated with lipidized cell wall monomers (i.e. lipidized cell wall monomer-associated genes), 851 genes that are uniquely associated with cuticular wax metabolites (cuticular wax-associated genes), and 379 genes that have associations with both lipidized cell wall monomers and cuticular waxes.

The ~1900 genes that were suggested to be cuticle-related by our joint statistical analyses were parsed into functional categories by performing two types of enrichment analyses: (i) GO enrichment of biological processes (GO-BP terms) (Ashburner *et al.*, 2000), which were further trimmed using REVIGO (Supek *et al.*, 2011); and (ii) enrichment into MapMan function bins (Schwacke *et al.*, 2019). The former descriptors identify the participation of individual genes in broad categories of biological processes, whereas the latter assign individual genes to different metabolic pathways.

Cross-referencing the results of MapMan analysis (Supplementary Table S5) and GO-BP analysis (Supplementary Tables S6–S8) identified 10 distinct metabolic pathways that were significantly enriched among the ~1900 putative cuticle-related genes. Specifically, 119 of the 699 lipidized cell wall monomer-associated genes (Supplementary Tables S5, S6) were significantly enriched with pathways associated with ‘Photosynthesis’, ‘Carbohydrate metabolism’, and ‘Coenzyme metabolism’ (Fig. 3). Forty-six of the 852 cuticular wax-associated genes were significantly enriched with three pathways: ‘Cellular respiration’, ‘Lipid metabolism’, and ‘Redox homeostasis’ (Fig. 3; Supplementary Tables S5, S7). Finally, 150 of the 379 genes that were associated with both lipidized cell wall monomers and cuticular waxes were significantly enriched with seven pathways: ‘Amino acid metabolism’, ‘Coenzyme metabolism’, ‘External stimuli response’, ‘Lipid metabolism’, ‘Photosynthesis’, ‘Protein homeostasis’, and ‘Vesicle trafficking’ (Fig. 3; Supplementary Tables S5, S8).

### Deduction of co-expression networks from the combination of cuticle-related gene clusters and multi-omics integration-based putative cuticle-related genes

**Fig. 5.**
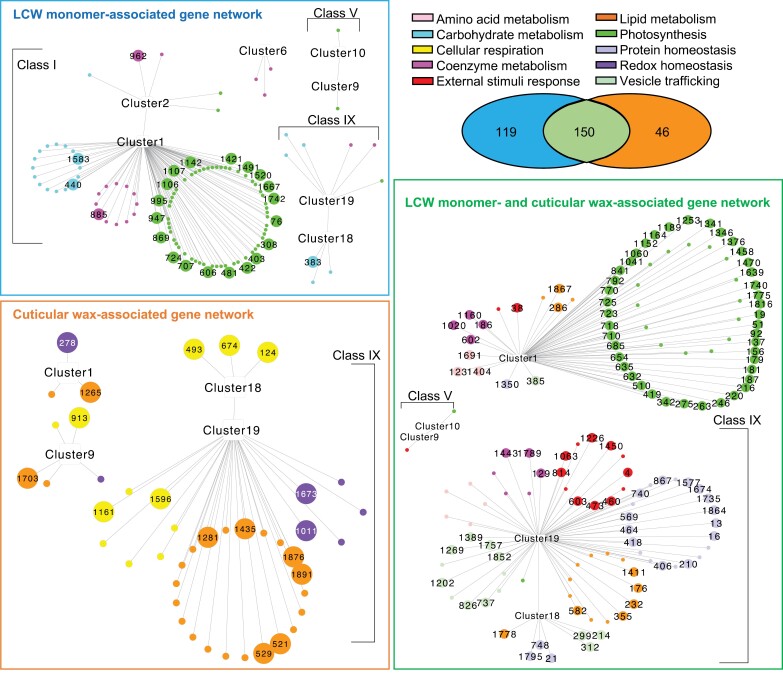
Co-expression gene networks identified by the combined weighted gene co-expression network analysis–random forest (WGCNA-RF) and multi-omics integration pipelines. The three gene networks incorporate the lipidized cell wall (LCW) monomer-associated genes (upper left panel), cuticular wax-associated genes (lower left panel), and LCW monomer- and cuticular wax-associated genes (right panel). Larger sized circles represent the hub genes identified by the WGCNA-RF pipeline. Gene identity is represented by the number in each circle and defined in Supplementary Table S4; the colors of these symbols identify different MapMan metabolic function bins.

Co-expression gene networks were constructed from 312 genes that resided within both the nine cuticle-related co-expression gene clusters (i.e. a total of 19 751 genes, including 715 hub genes) obtained from the WGCNA-RF (Supplementary Table S2) and the list of 315 putative cuticle-related genes identified by the multi-omics integration pipeline (Fig. 3; Supplementary Figs S8, S9; Supplementary Table S9). The three resultant gene co-expression networks include two networks that are specific for either lipidized cell wall monomers or cuticular waxes, and a third network that underlies both cuticle metabolomes (Fig. 5).

The lipidized cell wall monomer-associated gene network consists of 119 genes that were identified by the multi-omics pipeline, and these genes belong to seven WGCNA-identified co-expression clusters (Fig. 5). Most of the genes in this network (~87%) reside in Class I co-expression clusters (i.e. Clusters 1 and 2 of Fig. 4), wherein gene expression was highest in the leaf blades of the seedlings. The other genes in this network were distributed among Class IV (Cluster 6), Class V, and Class IX co-expression clusters (Figs 4, 5), with genes from Classes IV and V exhibiting the highest expression in the aerial organs compared with the roots. Moreover, 24 genes in this network were identified as hub genes within the WGCNA-identified co-expression clusters (Figs 3, 5). Collectively, the 118 genes associated with the lipidized cell wall monomer gene network were mapped to three metabolic pathways: ‘Photosynthesis’, ‘Coenzyme metabolism’, and ‘Carbohydrate metabolism’.

The cuticular wax-associated gene network that was identified by parallel analyses consists of 44 genes that were mapped to Class IX of the WGCNA-identified co-expression clusters (predominantly Cluster 19) (Fig. 5). Moreover, 17 genes in this network were also hub genes for co-expression Clusters 1, 9, 18, and 19, and most of these hub genes were annotated as being involved in either ‘Lipid metabolism’ or ‘Cellular respiration’.

Finally, the co-expression gene network that underlies both cuticle metabolomes (i.e. the lipidized cell wall monomers and the cuticular waxes) consists of 150 genes, which are parsed into two subnetworks that predominantly reside within the WGCNA-identified co-expression Clusters 1 and 19 (Figs 4, 5). Whereas the Cluster 1 sub-network is dominated by genes related to ‘Photosynthesis’, the Cluster 19 sub-network is composed of genes associated with ‘Protein homeostasis’, ‘Lipid metabolism’, ‘External stimuli response’, and ‘Vesicle trafficking’ (Fig. 5). Notably, 97 genes in this network were identified as hub genes within Clusters 1, 18, and 19. Seven of the 97 hub genes residing in this network are components of the ubiquitin–26S proteasome system that mediates post-translational regulation of gene expression.

Confidence in the associations between these gene networks and cuticle deposition is strengthened by the fact that 16 genes residing in the identified co-expression gene networks have previously been experimentally confirmed to participate in cuticle deposition. These include genes whose products are either directly involved in cuticle deposition, or are indirectly involved in this process by generating required precursors (Supplementary Table S9). For example, the ketoacyl-CoA synthase genes *KCS1* and *KCS24* and the ketoacyl-CoA reductase gene *KCR1* are components of the fatty acid elongation machinery that generates VLCFAs used for cuticular wax biosynthesis (Campbell *et al.*, 2019); *nsLTP2* and *ECHIDNA* gene products are involved in the transport of cuticular wax components to the extracellular space (Kim *et al.*, 2012; McFarlane *et al.*, 2014); *GLOSSY14* and the long-chain acyl-CoA synthetase *LACS1/CER8* gene products are required for cuticle biosynthesis (Zheng *et al.*, 2018); and the *FDL1-*encoded transcription factor acts as a positive regulator of cuticle biosynthesis (La Rocca *et al.*, 2015; Castorina *et al.*, 2020; X. Liu *et al.*, 2021). Genes that are involved in the generation of cuticle precursors include acyl carrier protein genes (*MTACP2* and *ACP*) (Souciet and Weil, 1992; Huang *et al.*, 2017; Fu *et al.*, 2020), three acyl-ACP desaturases (*SACD1*, *SACD10*, and *SACD11*) (Du *et al.*, 2016; Zhao *et al.*, 2019), and ATP-citrate lyase (*ACLA3*) (Fatland *et al.*, 2002). Products of these genes are collectively involved in either *de novo* fatty acid biosynthesis or fatty acid elongation that ultimately generates the VLCFA-CoA precursors of the cuticular waxes.

**Fig. 6.**
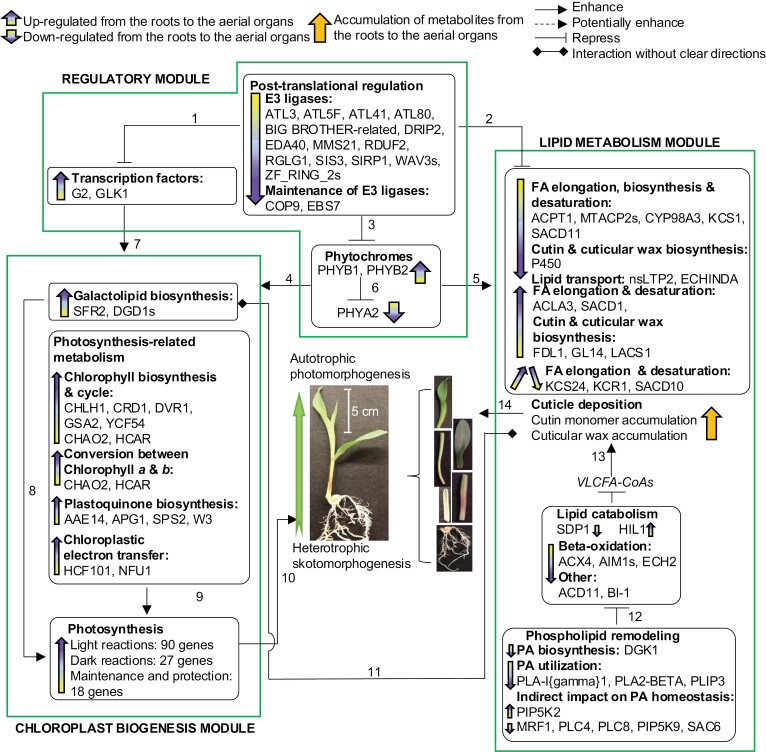
The regulatory program highlighting interactions between cuticle development and three functionality modules: a regulatory module, a lipid metabolism module, and a chloroplast biogenesis module. Numbered arrows identify interactions supported by literature-based evidence: (1) Tokumaru *et al.*, 2017; (2) Lü *et al.*, 2012; Lu *et al.*, 2013; Gil *et al.*, 2017; Wang *et al.*, 2018; H. Kim *et al.*, 2019; S. Liu *et al.*, 2021; Wu *et al.*, 2021; (3) J. Y. Kim *et al.*, 2017; Han *et al.*, 2020; (4) Legris *et al.*, 2019; (5) Qiao *et al.*, 2020; (6) Sheehan *et al.*, 2007; (7) Kobayashi *et al.*, 2014; Yeh *et al.*, 2021; (8) Rocha *et al.*, 2018; Fujii *et al.*, 2019; (9) Schwenkert *et al.*, 2010; Meguro *et al.*, 2011; Wang *et al.*, 2012; Reumann, 2013; Schlicke *et al.*, 2014; E.-H. Kim *et al.*, 2015; Kobayashi and Masuda, 2016; Pattanayak and Tripathy, 2016; E.-H. Kim *et al.*, 2017; Herbst *et al.*, 2018; Dmitrieva *et al.*, 2021; Satyanarayan *et al.*, 2021; (10) Shi *et al.*, 2020; (11) Xia *et al.*, 2009; Wan *et al.*, 2020; (12) S.-C. Kim *et al.*, 2019; Cai *et al.*, 2020; (13) Yeats and Rose, 2013; (14) Ingram and Nawrath, 2017.

The other genes that constitute these co-expression gene networks are primarily associated with two biological processes: (i) photosynthesis and photosynthesis-related metabolism, and (ii) lipid metabolism (Fig. 6; Supplementary Table S9). Photosynthesis and photosynthesis-related metabolism genes include those encoding components of Photosystems I and II [e.g. light-harvesting complex proteins (LHCs), photosystems I and II proteins (PSAs and PSBs)] (Teramoto *et al.*, 2001; Bressan *et al.*, 2018), key enzymes for carbon fixation [e.g. glyceraldehyde-3-phosphate dehydrogenases (GAPDHs) and phosphoenolpyruvate carboxylase (PEP1)] (Yang *et al.*, 2017), proteins that maintain and repair photosynthetic machinery (e.g. chaperone proteins) (Bai *et al.*, 2015), and proteins essential for chloroplast biogenesis (e.g. the transcription factors G2 and GLK1) (Kobayashi and Masuda, 2016; Rocha *et al.*, 2018; Fujii *et al.*, 2019; Yeh *et al.*, 2021) (Supplementary Table S9). Genes participating in lipid metabolism include those involved in triacylglycerol degradation (lipases such as *SDP1* and *MGAL5*) (Kim *et al.*, 2016; Fan *et al.*, 2017), fatty acid β-oxidation (e.g. acyl-CoA dehydrogenase *IBR3*, acyl-CoA oxidase *ACX4*) (Kim and Miura, 2004), and phospholipid metabolism and signaling (e.g. diacylglycerol kinase *DGK1*) (Wang *et al.*, 2006) (Supplementary Table S9).

Taken together, this suite of integrative analyses of transcriptomes and cuticle metabolomes identified not only known cuticle-related genes but also additional functionalities that either directly or indirectly impact cuticle deposition. These findings demonstrate the complexity of the gene networks underlying cuticle metabolomes, which involve the interaction between direct contributors to cuticle deposition and the genes belonging to biological processes co-occurring with cuticle deposition.

## Discussion

Maintaining global food production has become increasingly challenging over the past few decades. Although there are many causes for these challenges, a major contributor is global climate change that is the consequence of anthropogenic production of greenhouse gases (e.g. CO_2_, CH_4_, N_2_O and fluorinated gases), which are a result of the increased industrialization of human activities (Zaman *et al.*, 2021). Projecting forward, therefore, food crops will likely have to contend with increased abiotic and biotic stresses, including drought, salinity, solar radiation, elevated temperatures, pathogens, and pests (Gong *et al.*, 2014). These stresses may be analogous to the stresses that would have provided the selection pressure that led to the evolutionary appearance of the cuticle during the terrestrial colonization by land plants (Seale, 2020). As a water and gas barrier, the hydrophobic plant cuticle on aerial tissues (Riederer and Schreiber, 2001) and the suberin layer in the root periderm and endoderm (Hose *et al.*, 2001; Suresh *et al.*, 2022) are integral to protecting plants from these increasing stresses, particularly in maintaining water status. It is important to have detailed and integrated physiological, genetic, and biochemical insights into the processes that determine the deposition of different cuticles by crop plants.

Global agriculture is highly dependent on cereal crops, with maize being ranked third, after wheat and rice (Cooper *et al.*, 2014). As a biological system to study the cuticle, maize seedlings offer many advantages, including the availability of *glossy* mutants, first identified in 1928 (Hayes and Brewbaker, 1928), which affect the normal deposition of this material. Subsequent genetic and biochemical characterizations identified nearly 30 *glossy* alleles (Schnable *et al.*, 1994), and beginning in the 1990s many of these loci have been molecularly characterized (Moose and Sisco, 1994; Hansen *et al.*, 1997; Dietrich *et al.*, 2005; Lauter *et al.*, 2005; Sturaro *et al.*, 2005; Liu *et al.*, 2009; Li *et al.*, 2013, 2019; Zheng *et al.*, 2018; Alexander *et al.*, 2020). The parallel application of similar strategies with mutant collections in other plant species, such as Arabidopsis (Kunst and Samuels, 2009) and tomato (Petit *et al.*, 2021), have expanded the knowledge base of the mechanisms of cuticle deposition. These studies have largely validated the bifurcated structure of the metabolic process of cuticular wax deposition first suggested by the characterization of maize mutants (Bianchi *et al.*, 1985; Post-Beittenmiller, 1996). Namely, VLCFA products, generated by an endoplasmic reticulum-localized fatty acid elongase, are further metabolized through either a reductive pathway or a decarbonylative pathway, which collectively have the capacity to generate ~500 different cuticular wax components.

More recently, whole-genome research approaches have been employed to characterize the genetic architecture underlying cuticle deposition and function, broadening our knowledge of the genetic networks beyond the *glossy* genes. For example, gene co-expression networks in *glossy* mutants (Zheng *et al.*, 2018) have revealed aspects of transcriptional regulation of cuticle biosynthesis, and other studies have investigated the regulation of cuticle deposition by specific transcription factors (Castorina *et al.*, 2020; Qiao *et al.*, 2020; X. Liu *et al.*, 2021; Yang *et al.*, 2022). Genome- and transcriptome-wide association studies (Lin *et al.*, 2020, 2022) have further probed the genetic underpinnings and regulation of cuticular conductance. Even with these findings, the complete genetic network underlying cuticular wax biosynthesis and its regulation remains undefined. In contrast, there is even less knowledge concerning the genetic networks underlying the cutin component of the cuticle or of suberin, primarily because of the lack of mutants that affect the assembly of these lipid polymers.

In this study, we used a suite of approaches to explore the relationship between the transcriptome and biochemical phenotypes associated with the cuticle and suberin and, more broadly, with the lipidized cell wall, and ultimately identified gene networks associated with each of these metabolite phenotypes. This systems-based strategy enabled the exploration of the interrelationship between cuticular wax deposition and the formation of lipidized cell wall biopolymers with the developmental processes that establish the different seedling organs.

As previously reported by a number of studies (Bianchi *et al.*, 1985; Dietrich *et al.*, 2005), the cuticular waxes of the seedling organs are rich in products of the reductive branch of the cuticular wax biosynthesis pathway, namely, VLCFAs, fatty aldehydes and alcohols, with the majority (~90%) being of 32-carbon chain length. Furthermore, differences in the abundances of these constituents among the different organs are primarily quantitative, with the aerial organs, which have the largest exposure to the environment (i.e. the leaf blades), accumulating larger quantities of these metabolites, and coleoptiles and roots accumulating these metabolites at only ~10–20% of that abundance (Fig. 1).

The monomer composition of lipidized cell wall biopolymers is distinct from that of the cuticular waxes. In the seedling organs assessed in this study, the aliphatic fraction of these polymers was largely similar to a previous report of cutin composition in maize seedlings (Castorina *et al.*, 2020). Both studies detected high concentrations of ω-hydroxy-fatty acids, polyhydroxy-fatty acids, and VLCFAs, which were characterized in the cutin from a variety of plant species (Kolattukudy, 1980; Nawrath, 2002; Fich *et al.*, 2016; Bourgault *et al.*, 2020; Matschi *et al.*, 2020). Minor differences were observed between this study and that of Castorina *et al*. (2020) in the fatty acid (i.e. 18:2) and 2-hydroxy fatty acid content (Fig. 2), which could be attributed to one or more factors, including (i) the different genetic backgrounds used in the two studies, (ii) the depolymerization process, which released 2-hydroxy fatty acids from any residual membrane sphingolipids that remained after delipidization of the samples (Molina *et al.*, 2006), or (iii) the suberization of bundle sheath cells (Mertz and Brutnell, 2014; Danila *et al.,* 2021), which may also explain the high concentrations of aromatic monomers that were recovered (Fig. 2). Indeed, cuticular wax characterizations of the silks from ~30 maize inbred lines demonstrated that genetic background underlies substantial variation in wax composition (Dennison *et al.*, 2019), and the same might therefore be true for lipidized cell wall monomer composition. Finally, the aliphatic monomer composition observed for maize seedlings varies greatly from that of adult leaves, for which the cutin fraction has been found to be largely composed of ω-hydroxy fatty acids and α,ω-dicarboxylic acids, and also includes minor amounts of fatty acids (Bourgault *et al.*, 2020; Matschi *et al.*, 2020). These differences in aliphatic monomer composition between seedlings and adult leaves may reflect a role for leaf development in determining cutin composition.

A surprising finding in this study was the occurrence of a relatively large fraction of aromatic moieties in the lipidized cell wall biopolymers, including coumaric acid, ferulic acid, and caffeic acid (Fig. 2A). Such phenolics are commonly associated with suberin but are generally at lower abundance in cutin (Nawrath, 2002; Pollard *et al.*, 2008; Bourgault *et al.*, 2020), although phenolic-rich cutins have been found in a number of plant species, including the moss *Physcomitrella patens* (Caldicott and Eglinton, 1976; Girard *et al.*, 2012; Buda *et al.*, 2013; Kosma *et al.*, 2014; Renault *et al.*, 2017; Tomasi *et al.*, 2017, 2021; Razeq *et al.*, 2021). In Arabidopsis, an acyl-transferase was shown to esterify ferulic acid and ω-hydroxy-fatty acid monomers of the leaf cutin polyester (Rautengarten *et al.*, 2012), which is consistent with the occurrence of phenolics in the cutin polymer. Similarly, in the phenolic-rich moss cuticle, the aromatic cutin monomer caffeic acid is required to produce other aliphatic monomers (Renault *et al*., 2017). In adult-identity maize leaves, ferulic and coumaric acids are highly abundant in the lipid polymers extracted from whole leaves (Bourgault *et al.*, 2020), but are reduced to a low but quantifiable amount in the cutin fraction isolated from leaf blades wherein the vasculature has been removed (Borgault *et al.*, 2020). Therefore, the phenolic acid monomers detected in this study may be derived from several confounded sources, including the vascular tissues and/or the lipidized epidermal (aerial organs) or endodermal (roots) cell wall. Indeed, feruloyl and coumaroyl moieties are key features of cell walls in grass species, such as maize (Harris and Trethewey, 2010; Jung and Phillips, 2010; Hatfield *et al.*, 2017; Chandrakanth *et al.*, 2023).

In systems-based characterizations of biological processes, discerning relevance from coincidence can be challenging when extracting conclusions from complex datasets that integrate large numbers of molecular features that are altered by the biological status of the system. This is primarily because phenotype datasets are relatively small compared with the potential underlying genetic networks that determine the phenotype. For example, in this study, we investigated the relationships between cuticle composition (the phenotypes), measured as differences in the concentrations of ~100 metabolites, and differences in the gene expression program, measured as the accumulation levels of ~30 000 transcripts, in the context of 24 different biological states (i.e. six different organs of four different maize genotypes).

This complexity was harnessed by the application of two parallel statistical analytical pipelines, the WGCNA-RF and the multi-omics integration pipelines, which reduced the coincidental associations between the metabolome and the transcriptome (Fig. 3). Genes that were identified by the former pipeline were classified as hub genes (715 genes), and genes identified by the latter pipeline were identified as putative cuticle-related genes (~315 genes). These genes could be parsed into three functionalities that are associated with (i) cuticular wax biosynthesis, (ii) lipidized cell wall monomer synthesis (including cutin and suberin), or (iii) both cuticular wax and lipidized cell wall monomer biosynthesis (Fig. 3). These cuticle-associated genes include many transcriptional and post-translational regulators (e.g. bHLH, MYB, MADS, and WRKY family transcription factors, DNA/RNA-binding proteins, and components of the ubiquitin–26S proteasome system), which can be considered for future investigation of the regulatory mechanism for cuticle biosynthesis. We further refined this rich dataset by integrating the results from the WGCNA-RF approach and the multi-omics integration approach (Figs 4, 5), and thereby obtained metabolic modules that may directly or indirectly interact with cuticle deposition in young maize seedlings (Fig. 6); these are the focus of the remaining discussion.

Both pipelines identified genes that have previously been experimentally confirmed to be directly involved in cuticle biosynthesis (Fig. 6). These include genes involved in most steps of cuticular wax biosynthesis and deposition, including transcriptional regulation of the process, generation of upstream precursors to cuticle biosynthetic pathways, elongation of VLCFA-CoAs, and enzymatic components of the decarbonylative and reductive pathways of cuticular wax synthesis (Fatland *et al.*, 2002, 2005; Li-Beisson *et al.*, 2010; McFarlane *et al.*, 2014; La Rocca *et al.*, 2015; Campbell *et al.*, 2019; Castorina *et al.*, 2020; X. Liu *et al.*, 2021). In addition, four putative cuticle genes selected by our pipeline have also been identified by genome- and transcriptome-wide association studies as potentially involved in cuticle biosynthesis due to their role in cell wall biosynthesis (*PAE5*), vesicle trafficking (*Ypt/Rab-GAPs*), and regulation of cuticle development (*WRKY71*) (Lin *et al.*, 2022). The remaining cuticle-related genes identified by these pipelines can be parsed into three modules, which are associated with (i) chloroplast biogenesis, including galactolipid biosynthesis, chlorophyll metabolism, and photosynthesis; (ii) regulatory functions, including transcription factors, post-translational regulation, and phytohormones; and (iii) lipid metabolism, including processes such as cuticle synthesis, lipid catabolism, and phospholipid remodeling.

These latter findings suggest the involvement of chloroplast biogenesis and lipid metabolism in the generation of the seedling cuticle. However, because early seedling establishment involves the developmental transition from skotomorphogenesis to photomorphogenesis, many metabolic changes are associated with this transition from heterotrophic to autotrophic growth. For example, during this developmental transition there is a large increase in the photosynthetic capability of the aerial organs of the seedling, as evidenced by the increased expression of genes belonging to chloroplast biogenesis processes that are essential for the establishment and maintenance of photosynthetic machinery in the aerial organs. Concomitantly, there is down-regulation of genes involved in lipid metabolism (Fig. 6) that mobilize seed storage neutral lipids (i.e. triacylglycerol) and of fatty acid β-oxidation; these are processes that support the initial skotomorphogenic growth prior to the acquisition of photosynthetic capability by the emerging seedling. These observed associations between these processes and cuticle deposition could be a consequence of co-occurrence with the skotomorphogenesis–photomorphogenesis transition of the seedlings. However, a number of prior studies indicate a deeper, more mechanistic connection. For example, mutations that affect cuticular wax accumulation, such as *glossy* mutants in navel orange, also affect the biosynthesis of galactolipids, which are major components of the chloroplast membranes (Wan *et al.*, 2020). In Arabidopsis, mutations in the *ACP4* gene, which encodes an Acyl Carrier Protein (ACP) isozyme in the chloroplastic fatty acid synthase system, cause abnormal cuticle formation and a severe reduction in galactolipid accumulation (Xia *et al.*, 2009). Another prior study that supports our supposition of a connection between cuticle formation and photosynthesis and regulatory processes is the finding that mutations in phytochrome genes regulate cuticle development, specifically, the *phyA1 phyA2* and *phyB1 phyB2* double mutants of maize, and *phy* mutants in the moss *P. patens* (Qiao *et al.*, 2020). Finally, a number of prior studies have identified that targeted protein degradation, mediated by the ubiquitin–26S proteasome system, regulates cuticle deposition by degrading cuticular wax biosynthetic proteins or transcription factors that activate cuticular wax biosynthesis (Lü *et al.*, 2012; Lu *et al.*, 2013; Gil *et al.*, 2017; Wang *et al.*, 2018; S.-C. Kim *et al.*, 2019; S. Liu *et al.*, 2021; Wu *et al.*, 2021). Additionally, the E3 ligase SIS3, which is a key element of the ubiquitin–26S proteasome system, appears to be negatively regulated by the cuticle-controlling transcription factor FDL1 (S. Liu *et al.*, 2021). This study also identified other cuticle-related genes within the regulatory module that are associated with protein degradation, namely, two proteins that interact with E3 ligases (i.e. COP9 and EBS7), and these appear to control the development of the leaf epidermis and cuticle formation (Liu *et al.*, 2015; Xie *et al.*, 2020; Wu *et al.*, 2021).

In summary, using a suite of approaches for joint metabolome–transcriptome analysis and gene co-expression network analysis, this study has identified gene networks associated with cuticle metabolome compositional changes in six maize seedling organs that incorporate the transition from skotomorphogenesis to photomorphogenesis. These networks consist of genes with experimentally confirmed roles in cuticle deposition and putative cuticle-related genes, and, importantly, identify new suites of genes that either directly or indirectly impact cuticle formation during seedling development. The gene networks identified herein provide new gene targets for future molecular and genetic characterization to demonstrate their mechanistic roles in determining cuticle deposition. These gene networks will thereby provide routes for improving the defense properties of the cuticle that arms developing seedlings to withstand environmental challenges and maintain crop productivity in the face of global climate change.

## Supplementary data

The following supplementary data are available at *JXB* online.

Protocol S1. Multivariate models for joint analysis of the metabolome and transcriptome datasets.

Fig. S1. Delipidization of first and second leaf samples reduces the abundances of phosphatidylethanolamines, diacylglycerols and triacylglycerols, phosphatidic acids, and free fatty acids to the abundance of a reagent control.

Fig. S2. Comparison of the alkyl chain lengths of cuticular waxes, and lipidized cell wall monomers in maize seedling organs.

Fig. S3. Principal component analysis for cuticular waxes and lipidized cell wall monomers.

Fig. S4. PCA and tSNE visualization for transcriptome datasets.

Fig. S5. Four-way Venn diagrams that compare the differentially expressed genes between every pair of genotypes in each seedling organ.

Fig. S6. Heatmap representation of the number of differentially expressed genes between every pair of seedling organs in B73, Mo17, and the reciprocal hybrids.

Fig. S7. Similarity among eigengene expression for the 19 WGCNA-based co-expressed gene clusters (present in Fig. 4) as evaluated by hierarchical clustering and Pearson correlation-based adjacency heatmap.

Fig. S8. Heatmap visualization of the expression of the genes that were identified by the multi-omics integration analysis and were assigned to significantly enriched metabolic processes according to the enrichment analysis of Gene Ontology terms and MapMan function bins.

Fig. S9. Heatmap visualization of the expression of 135 ‘Photosynthesis’-pathway associated genes (participating in either the light-reactions or dark-reactions of photosynthesis) as identified by MapMan analysis.

Table S1. Delipidization analysis of seedling organ tissue.

Table S2. Lipidized cell wall monomers and cuticular waxes profiled from maize seedlings of B73, Mo17, and the reciprocal hybrids.

Table S3. Co-expressed gene cluster and the corresponding hub genes determined by WGCNA, and gene cluster-to-metabolome association analysis by a random forest regression model.

Table S4. Putative cuticle-related genes associated with the compositional changes of lipidized cell wall monomers and/or cuticular waxes as identified by the multi-omics integration pipeline.

Table S5. MapMan function enrichment test for putative cuticle-related genes that are associated only with lipidized cell wall monomer composition, only with cuticular wax composition, or with compositions of both lipidized cell wall monomers and cuticular waxes.

Table S6. Gene Ontology enrichment analysis for lipidized cell wall monomer-associated genes.

Table S7. Gene Ontology enrichment analysis for cuticular wax-associated genes.

Table S8. Gene Ontology enrichment analysis for lipidized cell wall monomer- and cuticular-wax associated genes.

Table S9. Putative cuticle-related genes that are annotated with significantly enriched metabolic processes that directly or indirectly interact with cuticle biosynthesis.

erae311_suppl_Supplementary_Protocol_S1_Figures_S1-S9

erae311_suppl_Supplementary_Table_S1

erae311_suppl_Supplementary_Table_S2

erae311_suppl_Supplementary_Table_S3

erae311_suppl_Supplementary_Table_S4

erae311_suppl_Supplementary_Table_S5

erae311_suppl_Supplementary_Table_S6

erae311_suppl_Supplementary_Table_S7

erae311_suppl_Supplementary_Table_S8

erae311_suppl_Supplementary_Table_S9

## Data Availability

The cuticle metabolome profiles are provided in Supplementary Table S2. RNA-seq datasets accompanied by detailed biosample metadata have been deposited in the NCBI Sequence Read Archive (accession PRJNA903105). All scripts used in this project are available in GitHub (https://github.com/ketingchen/MultiOmicsIntegration.git).

## Abbreviations:

ANOVA: analysis of variance
GO-BP: Gene Ontology terms for biological processes
HSD: honest significant difference
PCA: principal component analysis
PLS: partial least squares regression
RF: random forest
rGLM: random generalized linear model
sPLS: sparse partial least squares regression
tSNE: *t*-distributed stochastic neighbor embedding
VLCFA: very-long-chain fatty acids
WGCNA: weight gene co-expression network analysis
