# Supplementary material for: Cuticle development and the underlying transcriptome–metabolome associations during early seedling establishment
Source: J Exp Bot. 2024 Jul 20;75(20):6500–22. doi: 10.1093/jxb/erae311 (PMC11522977; doi:10.1093/jxb/erae311)
Supplement: erae311_suppl_Supplementary_Protocol_S1_Figures_S1-S9 [file erae311_suppl_supplementary_protocol_s1_figures_s1-s9.pdf]

Supplementary Protocol S1:

Multivariate models for joint analysis of the metabolome and transcriptome datasets

Data transformation:

The metabolome and transcriptome datasets were subjected to data transformation prior to multi-omics integration analyses by partial least square regression (PLS), sparse partial least square regression (sPLS), or random generalized linear model (rGLM). The transcriptome datasets were used as the predictor variables/features, and the metabolome datasets were the response variables. For PLS, the metabolome and transcriptome datasets were centered such that the average expression of each column was zero (de Jong, 1993). For sPLS and rGLM, the metabolome and transcriptome datasets were both centered and scaled such that the variance of expression in each column was one (Lê Cao et al., 2008).

Multivariate models

PLS

A flowchart that describes the steps of cuticle-related gene selection by PLS and sPLS is presented below. Both methods use the multivariate metabolome datasets as the response variables.

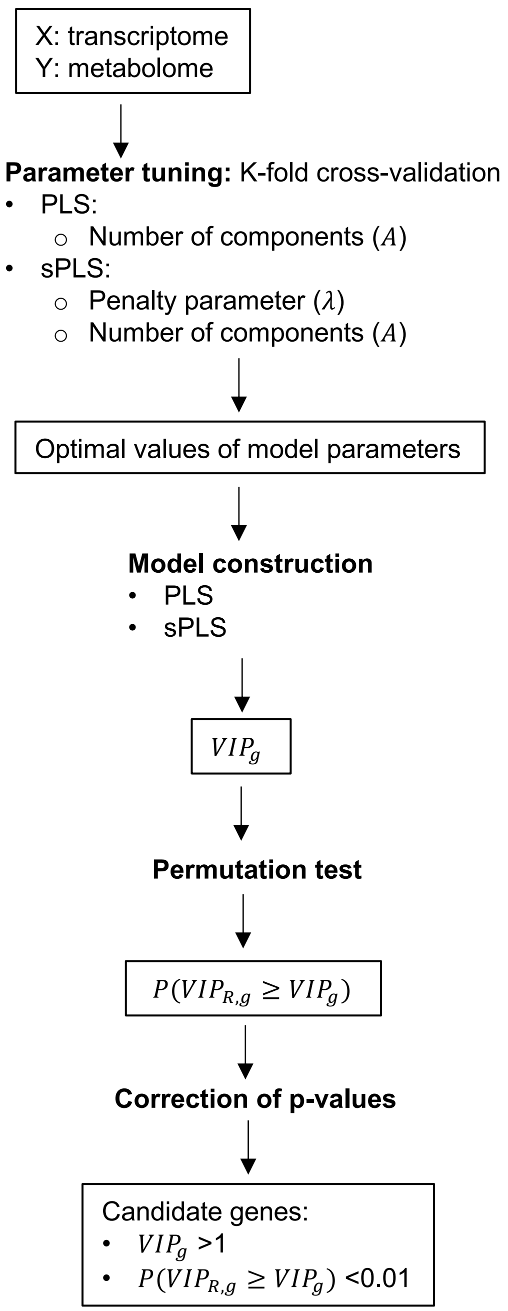

Main algorithm and calculation of feature importance

The scripts that perform PLS were written in the R language using the algorithm presented in de Jong (1993). This algorithm has been used to predict the clinical outcome with transcriptome

data (Pérez-Enciso and Tenenhaus, 2003) and has been included in the R package 'mixOmics' that is used for omics feature selection and data integration (Rohart et al., 2017).

Given the data matrices X $(n \times p)$ (i.e., transcriptomes) and Y $(n \times q)$ (i.e., metabolomes), PLS looks for the corresponding p and q- dimensional loading vectors u_a and v_a in each component a $(a = 1 \dots . A)$ to solve the optimization problem:

$$\max_{||u_a||=1, ||v_a||=1} Cov(X_{a-1}u_a, Y_{a-1}v_a)$$

where X_{a-1} and Y_{a-1} are the residual X and Y matrices after removing the effect of component $a - 1$, and when $a = 1$, X_0 and Y_0 are the original data matrices.

PLS measures the importance of each feature (i.e., gene) in predicting the metabolome compositions through the metric Variable Importance in Projection (VIP) that is calculated using the following equation (Akarachantachote et al., 2014):

$$VIP_{PLS} = \sqrt{p \times \frac{\sum_{a=1}^{A} \omega_a^2 \times SSY_a}{\sum_{a=1}^{A} SSY_a}},$$

where p is the total number of genes in the transcriptome data, ω_a is the weight of the gene in the PLS component a, SSY_a is the sum of square of the response (e.g., metabolome) explained by the component a, and A is the total number of components included in the model.

Selection of A:

A cross-validation strategy was used to identify the optimal value of A to include in the model. In detail, the joint metabolome and transcriptome datasets were first divided into K subsets (i.e., K folds), for K typically an integer between 2 and 10. Then, $K - 1$ folds of dataset were used as the training set for the model construction, and the remaining one fold was used as the validation set to evaluate the model's predictive performance by three metrics: 1) predictive R^2; 2) root mean square error (RMSE), and 3) mean absolute percent error (MAPE). The

procedures described above were repeated until every fold of data was used as the validation set (i.e., K repetitions). Next, the entire cross-validation was repeated three times, each time with a different random partition. The evaluating metrics (predictive R^2, RMSE, and MAPE) were averaged among all repetitions (i.e., $3 \times K$). For our results, the chosen A produced an optimal model performance in at least two metrics.

<u>Permutation test</u>

To identify the features most relevant to the response variables, features g with VIP score $VIP_g > 1$ are typically selected (Cocchi et al., 2018). However, it is well-known that 1 is not always the ideal threshold, and some non-relevant features can achieve $VIP_g > 1$ (Tran et al., 2014; Farrés et al., 2015). To further ensure that VIP scores achieving the threshold are biologically meaningful, we permuted metabolome datasets relative to the associated transcriptome datasets to disrupt the biological signal driving gene-metabolite correlations. The background VIP level for an individual gene g ($VIP_{R,g}$) was estimated by a PLS model on the permuted datasets. The probability of obtaining a background $VIP_{R,g}$ exceeding the observed VIP_g in the absence of biological signal was computed as:

$$P\left(VIP_{R,g} \geq VIP_g\right) = \frac{1 + \sum_{b=1}^{B} I(VIP_{R,g}^{b} \geq VIP_g)}{B + 1}$$

where B represents the number of permutations and $VIP_{R,g}^{b}$ is the background VIP in the bth permutation. The resultant probabilities were next corrected among all genes to control a false discovery rate <5% according to Benjamini and Hochberg (1995). A gene was considered as a potential determinant of metabolome compositions when VIP_g >1 and the associated $P\left(VIP_{R,g} \geq VIP_g\right)$ <0.01.

sPLS

The script that performs sPLS was written in the R language based on the scripts for PLS, with modifications. Herein a soft-thresholding L1 penalty function on the elements of u_a was included, i.e., $g_\lambda(u_a) = sign(u_a)(|u_a| - \lambda)_+$, as described in Lê Cao et al. (2008).

A "bagging" strategy was appended to the original sPLS method by generating 1,000 versions of the original datasets (i.e., 1000 bags) through bootstrapping samples and selecting a random subset of $\sqrt{p}$ features (i.e., genes), following the steps delineated in Song et al. (2013) and presented in the chart below.

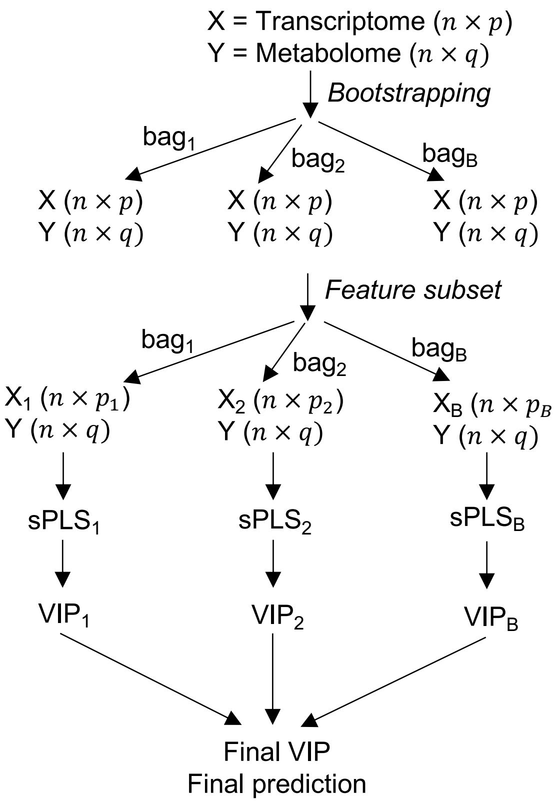

An sPLS model was constructed for each bag, following the method described in Lê Cao et al. (2008), which generates a VIP for each gene included in the bag. The final VIP_g for gene g was averaged over all bags containing this gene. The final prediction of metabolome

compositions was averaged across the predictions of each bag. The probability for background $VIP_{R,g}$ exceeding the observed VIP_g, $P(VIP_{R,g} \geq VIP_g)$, was calculated by repeating the entire procedure (i.e., bagging and construction of sPLS models) on the permutation simulations described in the earlier section.

The parameters that were tuned for sPLS include the penalty parameter λ and the number of sPLS components A. A cross-validation strategy was used to identify the optimal combination of these parameters similar to the strategy described for PLS. In particular, the penalty parameter λ was evaluated at different percentiles among the absolute values of elements in u_a from the 20th to the 80th percentile at every 10-percentile interval.

rGLM

rGLM was performed using the randomGLM function in R/randomGLM package (Song et al., 2013) with the following modifications. First, prior to rGLM, genes to be included in the model were selected by a preliminary PLS evaluation with the transcriptome datasets both centered and scaled. Only the genes with a resultant VIP >1 were subjected to the next round of selection by rGLM. The rGLM method is a forward selected generalized linear model (GLM) combined with a bagging strategy that is described in the section on sPLS (Song et al., 2013). In rGLM, a gene is identified as a cuticle-related gene if this gene has been selected by the GLM in at least one bag of data. The total number of bags wherein a gene is selected (N_g) is thus considered a measure of variable importance (Song et al., 2013).

Similar to PLS and sPLS, we evaluated the background count for each gene g ($N_{R,g}$) in datasets that were created by permuting the transcriptome datasets across biological samples and

therefore were devoid of correlations caused by biological signals. The probability of obtaining a selection count greater than the N_g observed for gene *g* is computed as:

$$P\left(N_{R,g} \geq N_g\right) = \frac{1 + \sum_{b=1}^{B} I(N_{R,g}^{b} \geq N_g)}{B + 1}$$

where B represents the number of permutations and $N_{R,g}^{b}$ is the background count N_g from the *b*th permutation.

The resultant probabilities were next corrected among all genes to control a false discovery rate <5% according to Benjamini and Hochberg (1995). A gene was considered as a potential determinant of metabolome compositions when $N_g \geq 1$ and the associated $P\left(N_{R,g} \geq N_g\right) < 0.01$.

REFERENCES

Akarachantachote N, Chadcham S, Saithanu K (2014) Cutoff threshold of variable importance in projection for variable selection. International Journal of Pure and Applied Mathematics. doi: 10.12732/ijpam.v94i3.2

Benjamini Y, Hochberg Y (1995) Controlling the False Discovery Rate: A Practical and Powerful Approach to Multiple Testing. Journal of the Royal Statistical Society: Series B (Methodological) **57**: 289–300

Farrés M, Platikanov S, Tsakovski S, Tauler R (2015) Comparison of the variable importance in projection (VIP) and of the selectivity ratio (SR) methods for variable selection and interpretation. J Chemom **29**: 528–536

de Jong S (1993) SIMPLS: An alternative approach to partial least squares regression. Chemometrics and Intelligent Laboratory Systems **18**: 251–263

Lê Cao K-A, Rossouw D, Robert-Granié C, Besse P (2008) A Sparse PLS for Variable Selection when Integrating Omics Data. Stat Appl Genet Mol Biol. doi: 10.2202/1544-6115.1390

Pérez-Enciso M, Tenenhaus M (2003) Prediction of clinical outcome with microarray data: a partial least squares discriminant analysis (PLS-DA) approach. Hum Genet **112**: 581–92

Rohart F, Gautier B, Singh A, Lê Cao K-A (2017) mixOmics: An R package for ‘omics feature selection and multiple data integration. PLoS Comput Biol **13**: e1005752

Song L, Langfelder P, Horvath S (2013) Random generalized linear model: a highly accurate and interpretable ensemble predictor. BMC Bioinformatics **14**: 5

Tran TN, Afanador NL, Buydens LMC, Blanchet L (2014) Interpretation of variable importance in Partial Least Squares with Significance Multivariate Correlation (sMC). Chemometrics and Intelligent Laboratory Systems **138**: 153–160

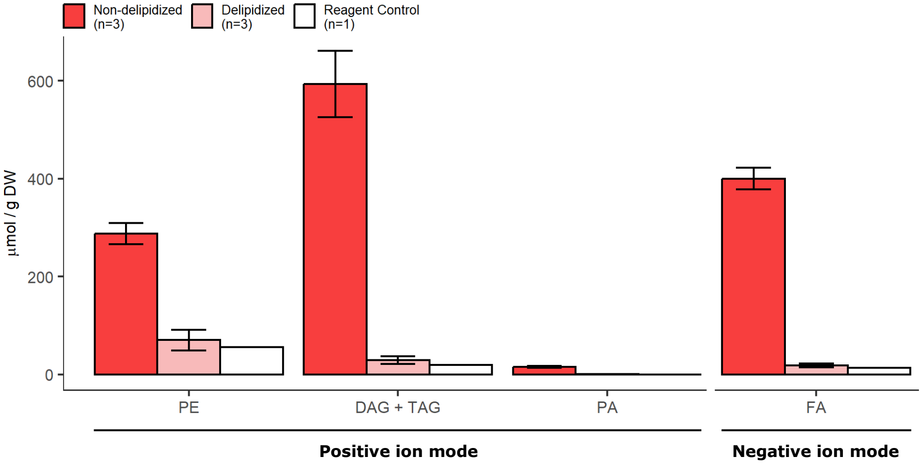

Supplementary Fig. S1: Delipidization of first and second leaf samples reduces phosphatidylethanolamines (PE), diacylglycerols (DAG) and triacylglycerols (TAG), phosphatidic acids (PA), and free fatty acids (FA) abundances to that of a reagent control. Lipid abundances were quantified by LC-MS/MS, ran in either positive or negative ion mode.

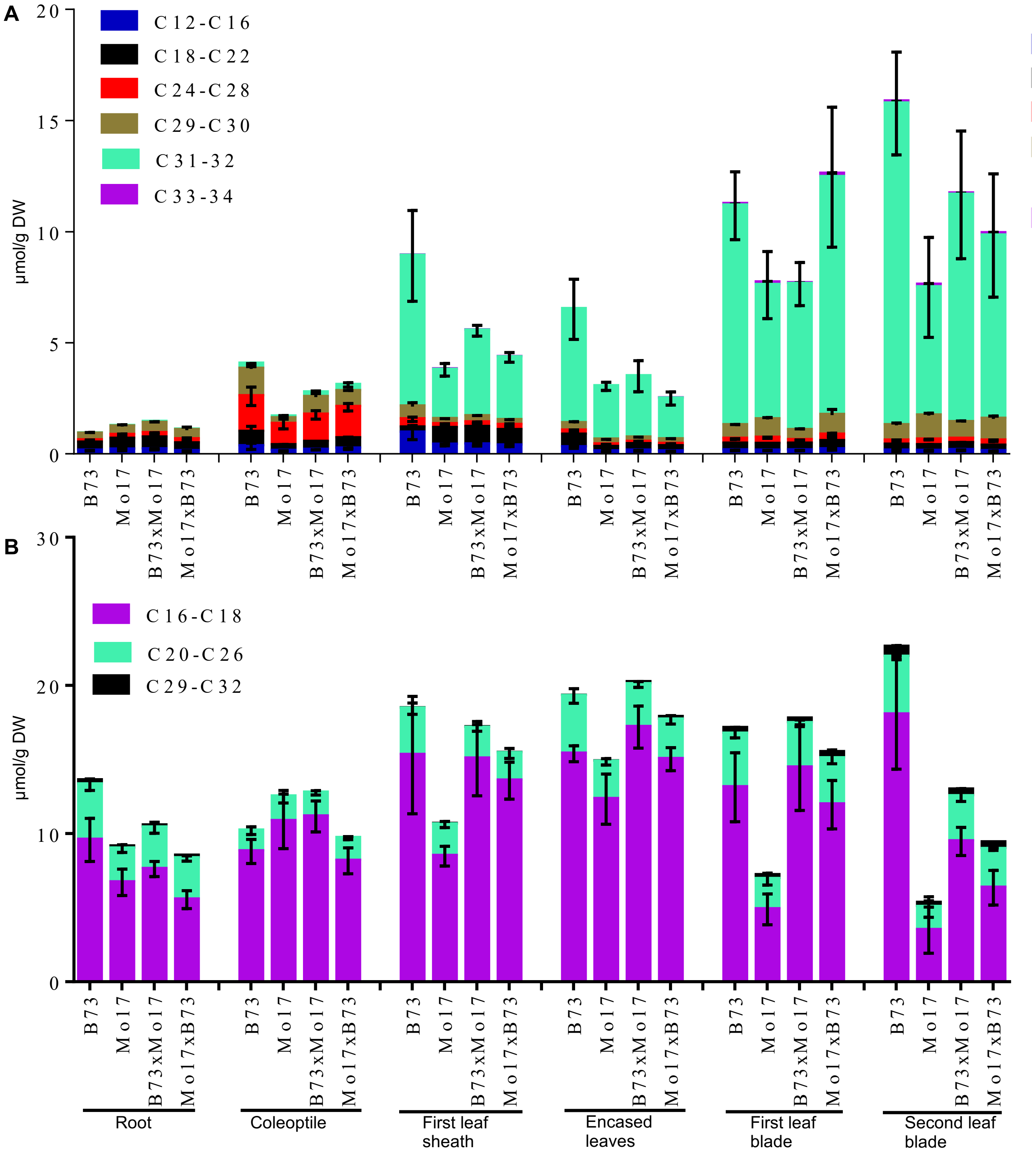

Supplementary Fig. S2: Comparison of the alkyl chain lengths of cuticular waxes (A), and lipidized cell wall monomers (B) in maize seedlings organs.

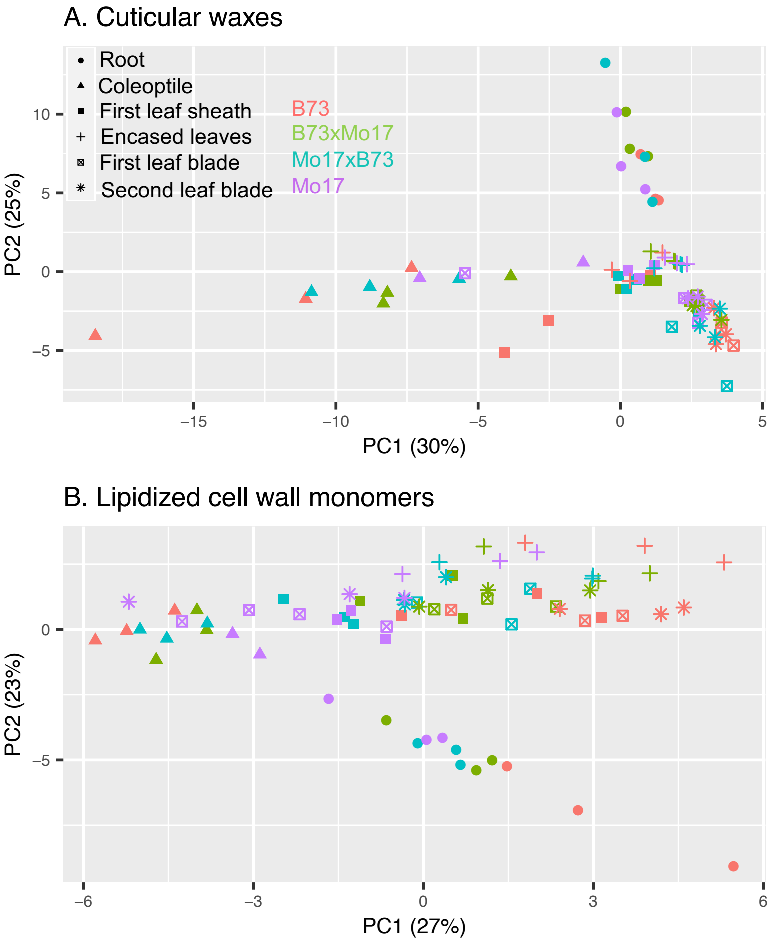

Supplementary Fig. S3: Principal component analysis (PCA) for cuticular waxes and lipidized cell wall monomers. Symbol color denotes genotype and symbol shape denotes seedling organ. The percentages listed represents the percent of variance explained by that PC. Abbreviations: PC, principal component; PCA, principal component analysis.

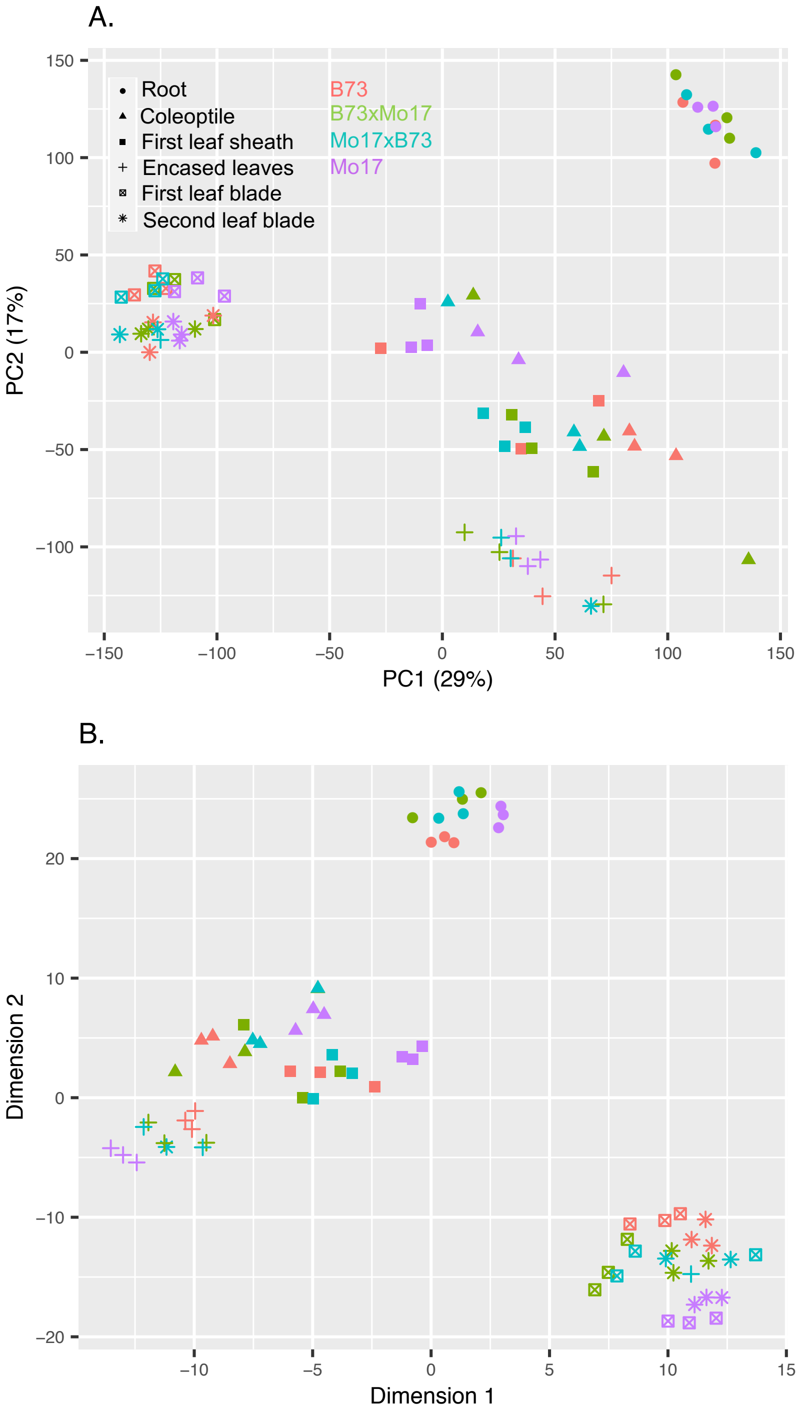

Supplementary Fig. S4: PCA (A) and tSNE (B) visualization for transcriptome datasets. The percentages listed in (A) represents the percent of variance explained by that PC. Abbreviations: PC, principal component; PCA, principal component analysis; tSNE, t-distributed stochastic neighbor embedding.

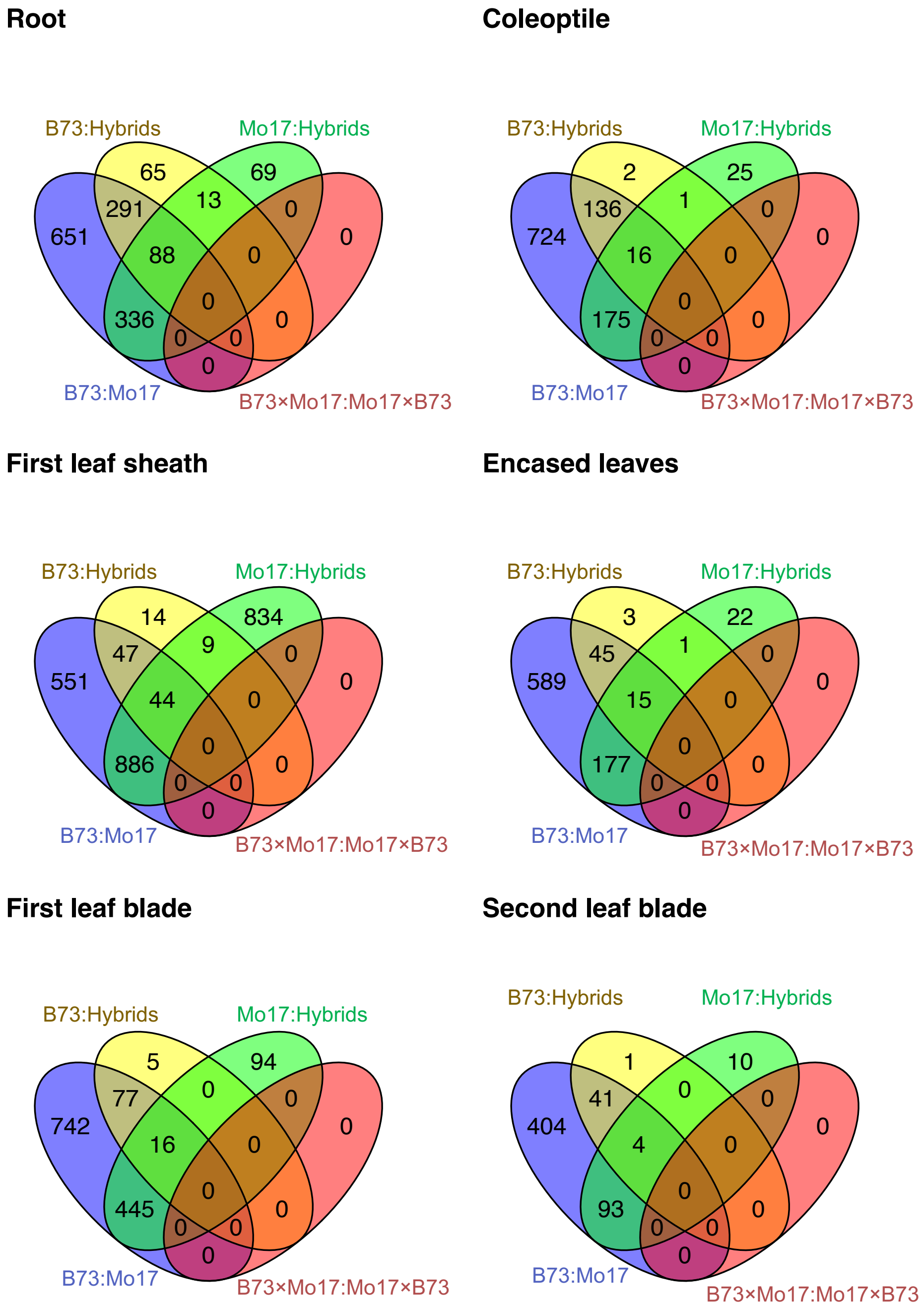

Supplementary Fig. S5: Four-way Venn diagrams that compare the differentially expressed genes between every pair of genotypes in each seedling organ.

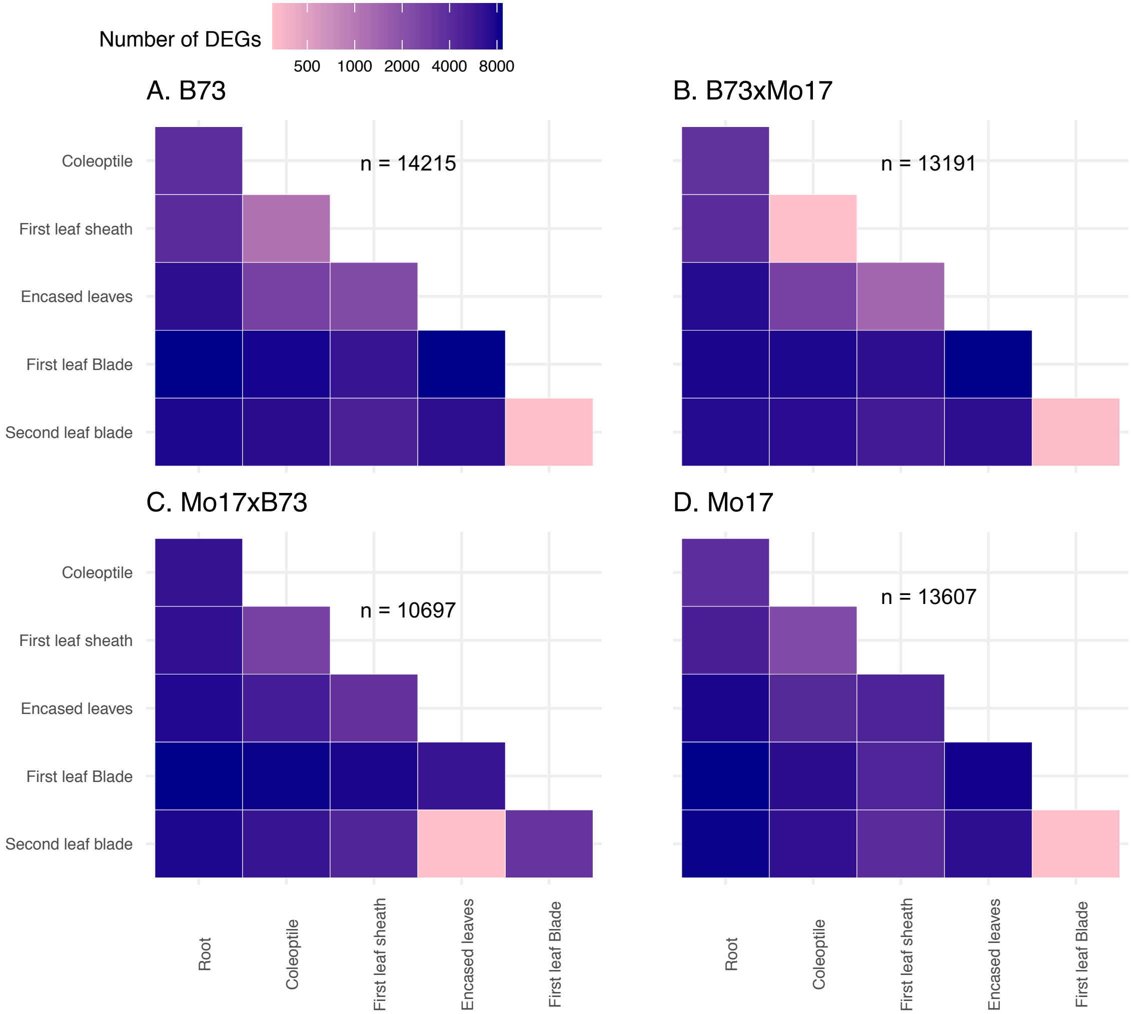

Supplementary Fig. S6: Heatmap representation of the number of differentially expressed genes between every pair of seedling organs in B73, Mo17, and the reciprocal hybrids. The total number of non-redundant differentially expression genes is indicated for each genotype (n).

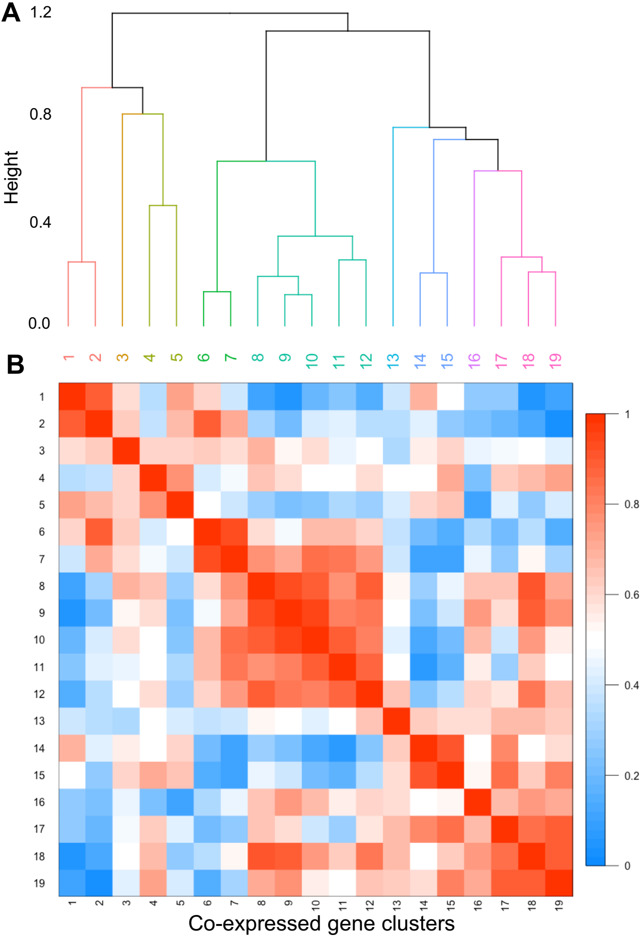

Supplementary Fig. S7: Similarity among eigengene expression for the 19 WGCNA-based co-expressed gene clusters (present in Fig. 4) as evaluated by hierarchical clustering (A) and Pearson-correlation based adjacency heatmap (B). Abbreviation: WGCNA, weighted gene co-expression network analysis.

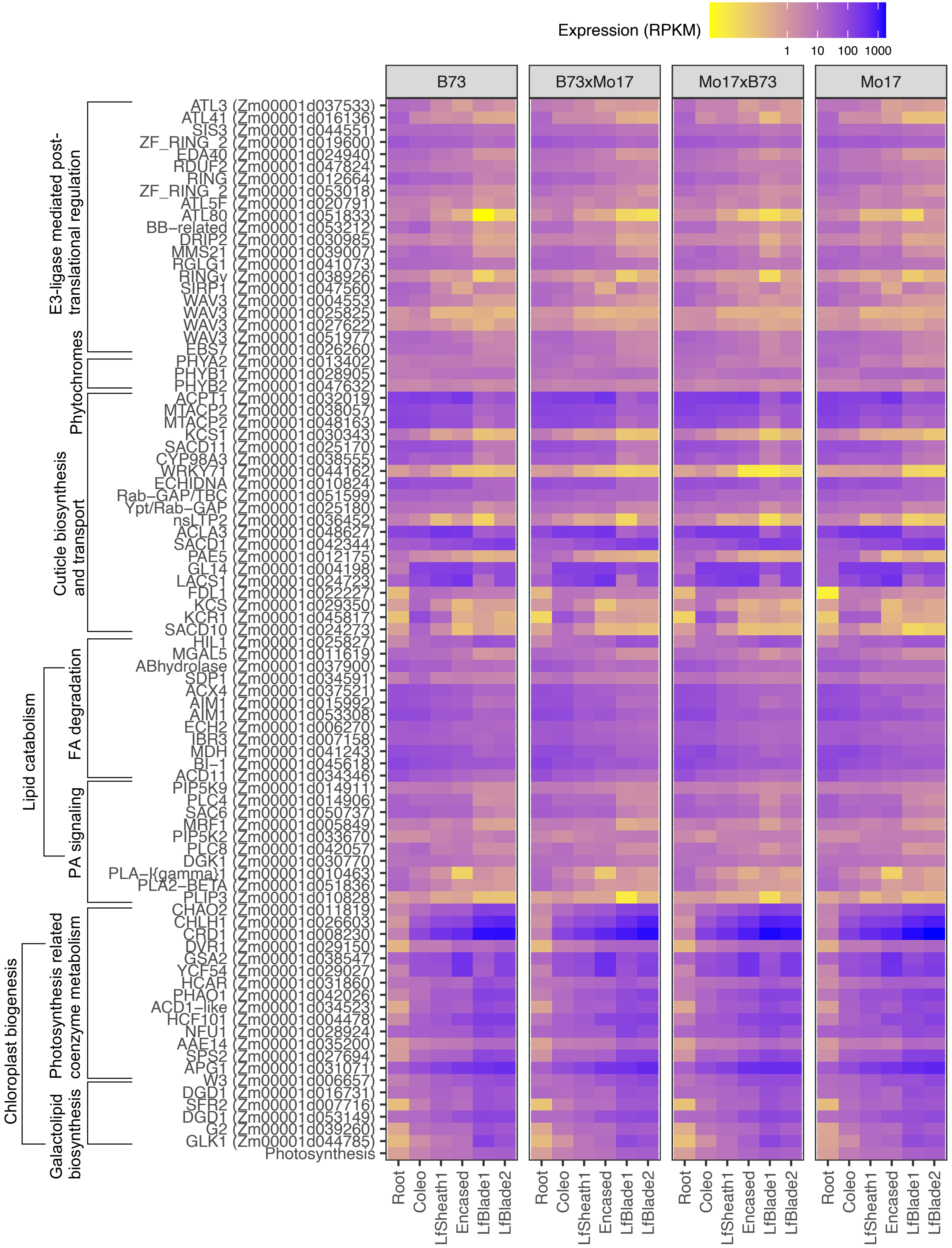

Supplementary Fig. S8: Heatmap visualization of the expression of the genes that were identified by the multi-omics integration analysis and were assigned to significantly enriched metabolic processes according to the enrichment analysis of gene ontology (GO) terms and MapMan function bins. The expression of 135 photosynthesis genes is summarized and presented as a single eigengene, labeled "Photosynthesis" at the bottom of the plot. The expression of these photosynthesis genes are separately presented in Supplemental Fig. S9. Abbreviations: Coleo, coleoptile; LfSheath1, fist leaf sheath; Encased, encased leaves; LfBlade1, first leaf blade; LfBlade2, second leaf blade; GO, gene ontology.

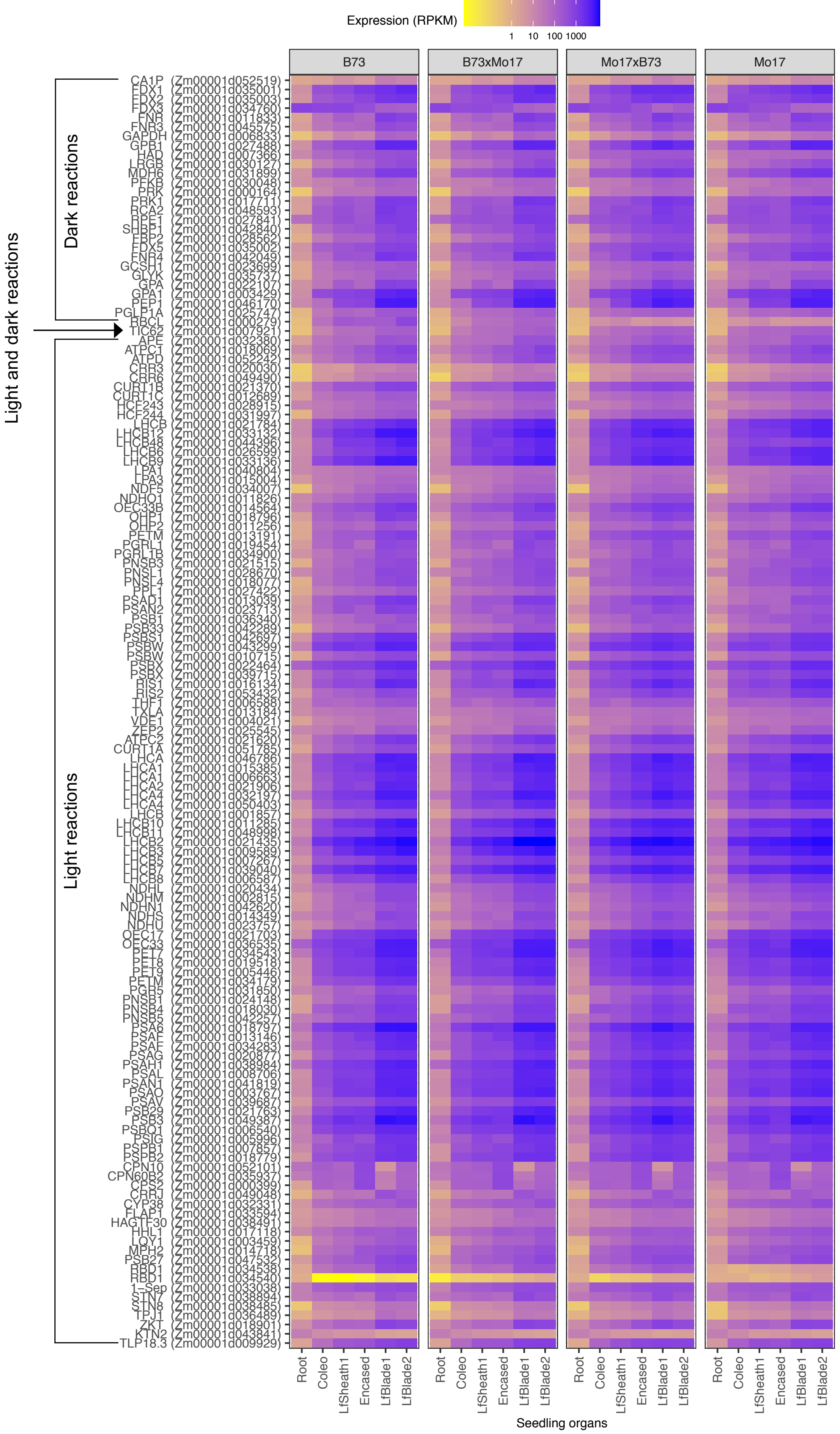

Supplementary Fig. S9: Heatmap visualization of the expression of 135 "Photosynthesis"-pathway associated genes (participating in either the light-reactions or dark-reactions of photosynthesis) as identified by MapMan analysis.
